# Toll-like receptor signaling outcome is determined by the stoichiometry of the endogenous TRIFosome

**DOI:** 10.1126/sciadv.aeb9507

**Published:** 2026-03-06

**Authors:** Martin C. Moncrieffe, Prasanna Suresh, Joe Boyle, Yuhao Cui, Bharti Nawalpuri, Brett Verstak, Yu P. Zhang, Ziwei Zhang, Marcus Taylor, Edward H. Egelman, Nicholas Gay, David Klenerman, Clare Bryant

**Affiliations:** ^1^ Department of Biochemistry, University of Cambridge, Cambridge, UK.; ^2^ Yusuf Hamied Department of Chemistry, University of Cambridge, Cambridge, UK.; ^3^ Department of Veterinary Medicine, University of Cambridge, Cambridge, UK.; ^4^ Department of Medicine, University of Cambridge, Cambridge, UK.; ^5^ Max Planck Institute for Infection Biology, Berlin, Germany.; ^6^ Department of Biochemistry and Molecular Genetics, University of Charlottesville, Charlottesville, VA, USA.

## Abstract

Toll-like receptors (TLRs) drive innate immunity via assembly of macromolecular signal transduction platforms [supramolecular organizing centers (SMOCs)] coordinated by adaptor proteins such as Toll/interleukin-1 receptor (IL-1R) domain–containing adaptor-inducing interferon-β (TRIF), but whether oligomeric TRIFosomes form is unknown. Here, using cryo–electron microscopy and biophysical characterization of full-length TRIF in vitro, we show that it forms filamentous oligomers, which associate with the TRIF signaling partners receptor interacting protein 1 (RIP1) and RIP3 kinases, suggesting that oligomeric TRIFosomes could form. Endogenous TRIF, however, is predominantly monomeric in the absence of ligand, only forming TRIFosome oligomers in macrophages after stimulation of TLR4 or TLR3 when large, macromolecular signaling complexes form. TRIFosomes are fully formed 45 min after TLR3 or 60 min after TLR4 stimulation, commensurate with activation of nuclear factor κB in these cells. TLR3/4 activation triggers rapid interferon signaling prior to TRIFosome formation through monomeric TRIF, unexpectedly suggesting that a macromolecular platform of TRIF is not required to drive this signaling pathway. Collectively, these data show TRIFosome macromolecular platform formation and, unexpectedly, that TLR signaling can be SMOC-independent in addition to being SMOC-dependent.

## Teaser

A previously entirely unknown mode of TLR signal transduction is elucidated.

## INTRODUCTION

Pattern recognition receptors of the innate immune system detect conserved microbial patterns to activate an immune response against pathogens (*1*). These receptors signal by forming macromolecular protein complexes [supramolecular organizing centers (SMOCs)] to trigger inflammation (*2*). Apart from Toll-like receptor 3 (TLR3), all TLRs signal through the adaptor protein myeloid differentiation primary response protein 88 (MyD88), which forms the oligomeric MyDDosome signaling platform (*3*, *4*). The formation of MyDDosomes triggers the transcription of inflammatory proteins by promoting nuclear translocation of the proinflammatory transcription factor nuclear factor κB (NF-κB) (*5*). TLR4 also uses a second adaptor protein Toll/interleukin-1 receptor (IL-1R) domain–containing adaptor-inducing interferon-β (TRIF), shared with TLR3, to drive both type I interferon (IFN) and NF-κB signaling (*6*–*8*). A TRIF-based macromolecular signaling platform, the “TRIFosome” (*9*), analogous to the MyD88 MyDDosome SMOC, has been proposed to coordinate TRIF-dependent signaling, but evidence for its formation remains elusive.

Here, we characterize the structure of TRIF and show that it forms oligomers in vitro that associate with the putative TRIFosome signaling proteins receptor interacting protein kinase 1 (RIPK1) and RIPK3, supporting the potential of TRIF to form a SMOC. We endogenously tagged TRIF in macrophages with the fluorescent protein mStayGold, and, after stimulation of TLR4 or TLR3, we used single-molecule fluorescence analysis to show the formation of large complexes within cells, which associate with RIPK1 identifying the “TRIFosome” signaling platform. The TRIFosome is important for NF-κB activation but, unexpectedly, not for triggering TRIF-dependent IFN signaling, which occurs from monomeric or transient di- or trimeric TRIF complexes. Collectively, these data suggest that TLR-driven TRIF signaling is modular: first signaling from monomer or transient lower-order complexes to trigger type I IFN production and then progressing to form stable, complex signaling platforms over time.

## RESULTS

### TRIF forms filamentous oligomers in vitro

**Fig. 1.**
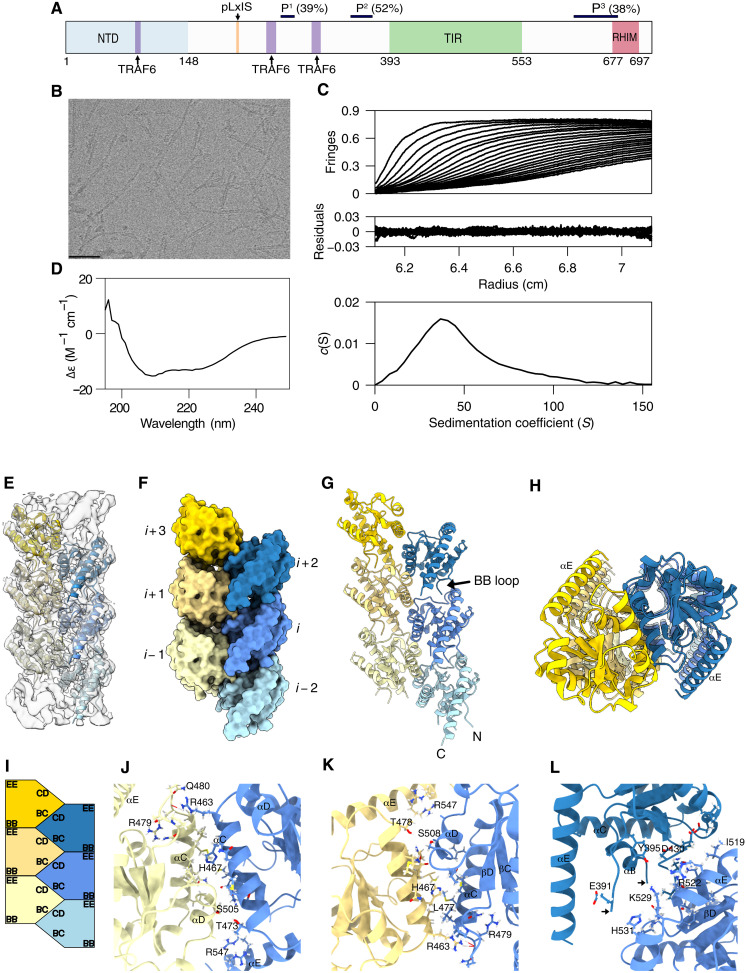
TRIF forms helical filaments. (**A**) TRIF consists of an NTD followed by an unstructured segment before the TIR domain that contains two proline rich regions—P^1^ (39%) and P^2^ (52%)—several interaction sites for TNF receptor associated factor (TRAF6) and a pLxIS motif, which is phosphorylated by Tank binding kinase (TBK1) to activate interferon regulatory factor 3 (IRF3) signaling. A third proline rich region (P^3^) and a RHIM complete the annotated UniProt domain boundaries. (**B**) Cryo-EM image of the filaments formed by TRIF. Scale bar, 50 nm. (**C**) Sedimentation velocity profiles, residuals, and the *c*(*S*) distribution for TRIF filaments. The *c*(*S*) distribution has a peak at 37*S*, but its width suggests variable filament lengths. (**D**) Far-ultraviolet circular dichroism spectrum is indicative of α-helical secondary structure (minima at 222 and 208 nm). (**E**) Cryo-EM reconstruction and a hexameric segment of the final model. (**F**) Surface representation showing subunit relationships. (**G** and **H**) Cartoon representation and a view along the helical axis. The third α helix (αC) is centrally located and almost perpendicular to the helical axis; the biologically important BB loop is shown. (**I**) Interacting residues in the TRIF-TIR. The *i*:*i* − 1, (**J**) and *i*:*i* + 1 (**K**) interfaces involve residues in αC and αD. The *i*:*i* ± 2 interface (**L**) consists of interactions with the BB loop of the *i* + 2 subunit and αE of subunit *i* or, alternatively, αE of subunit *i* − 2 and the BB loop of subunit *i*. Arrows denote missing residues of the BB loop.

To investigate whether TRIF forms oligomeric signaling complexes, we first determined its structural organization using cryo–electron microscopy (cryo-EM) and complementary biophysical techniques. TRIF contains a central Toll/IL-1R homology (TIR) domain flanked by a structured N-terminal domain (NTD) and a C-terminal region, which contains a RIP homotypic interaction motif, or RHIM (Fig. 1A). RHIM domains are characterized by the presence of a conserved tetrapeptide (VQIG) (*10*). The presence of the fibril-forming RHIM and, potentially, the TIR domain suggests that TRIF, like MyD88, RIPKs, and the TLR sorting adaptor MyD88-like TIR domain–containing adaptor protein (MAL/TIRAP), may form filaments. Full-length TRIF spontaneously assembles into functional helical filaments (fig. S1) that have a peak sedimentation coefficient of 37 *S* (Fig. 1, B to D). Filament assembly is dependent on the TIR and RHIM motifs. R4A mutation of TRIF in the RHIM tetrapeptide (R4A mutant) results in shorter and morphologically distinct filaments compared to those formed by wild-type (WT) TRIF. The TRIF BB-loop P434H mutation does not prevent formation of filaments. A splice isoform of TRIF, TRIS, in which the TIR domain is deleted, self-associates into filaments, whereas TRIS with the RHIM R4A mutation (in which the four conserved RHIMs, 687-VQLG-690, are mutated to alanine residues) does not form oligomers (figs. S2 to S4) (*11*).

### Structural analysis of TRIF filaments

Analysis of the averaged power spectrum obtained on TRIF filaments (fig. S5) revealed P2_1_ symmetry with an axial rise of ~16 Å and an azimuthal rotation angle of 180°. Iterative helical real-space refinement (*12*) as implemented in CryoSPARC (*13*) calculated a 4.2-Å map with refined helical parameters of 16.1 Å and 178.3° (table S2). Examination of the cryo-EM map revealed that full-length TRIF could not be accommodated in the coulombic map, implying that the observed filaments likely form from either homo- or heterotypic association of domains within TRIF. An AlphaFold model of TRIF (*14*) predicts an unusually long (65 amino acids) fifth α helix (αE) at the C terminus of the TIR domain, which is seen in the cryo-EM map. A trimmed version of the predicted TIR (fig. S6) domain was fitted into the map using MOLREP (*15*) and refined using PHENIX (*16*). Several rounds of refinement and model building (*17*) were performed, and six copies of the TIR were docked into the cryo-EM map and refined (Fig. 1E). Superposition of the coordinates for the AlphaFold and docked TIR domain, along with C_α_-atom root mean square deviation (RMSD), is shown in fig. S6 (B and C).

The architecture of the TRIF filament consists of two almost parallel protofilaments (Fig 1, E to H), and the structures of all copies are identical with an all-atom RMSD of 0.001 Å. Each TIR domain, 𝑖, makes predominantly hydrophobic contacts with four neighboring subunits, 𝑖 − 2, 𝑖 − 1, 𝑖 + 1, and 𝑖 + 2 (Fig. 1F), and the interactions are broadly similar to those observed between the intrastrand subunits of the MAL^TIR^ filament (*18*). Of these, the 𝑖:𝑖 − 1 interface (Fig. 1, I and J) is the largest burying 457 Å^2^. Residues in αC of subunit 𝑖 (R463, L464, H467, N470, Q471, M474, S475, and T478) interact with residues in αC of the 𝑖 − 1 subunit (M473, M474, N476, L477, and T478), αD (S501, D502, S505, L506, and L507), and also the αC-βD loop (R479, Q480, S482, G481, and P483) and αD-βE loop regions (S508, G509, and L510). There are also contacts between residues at the start of the αB (Q443) of subunit 𝑖 with residues in αD (S505, L506, and S508) of the 𝑖 + 1 subunit. The R547 (αE) of subunit 𝑖 makes contacts with S501 and D502 in αD of the 𝑖 + 1 subunit. Hydrogen bonds between the side-chain amines of R463 (𝑖), with the carbonyl of R479, also help to stabilize this interface. The marginally smaller 𝑖:𝑖 + 1 interface (Fig. 1K) buries 450 Å^2^ and arises from interactions between residues in the third helix (L466, N470, M473, M474, N476, and L477) and the αC-βD loop (Q480, G481, and P483) of subunit 𝑖, with residues of αC on the 𝑖 + 1 subunit (R463, L464, and H467). There are also contacts between residues in the fourth helix (αD) (S501, S505, L506, and L507) and the αD-βE loop (S508, G509, and L510) of subunit 𝑖 and residues in second (Q443), third (R463, L464, H467, Q471, and M474), and fifth helices (R547) of the 𝑖 + 1 subunit. This interface is stabilized by hydrogen bonds between the carbonyl of R479 (𝑖) and the side-chain amine groups of R463 (𝑖 + 1). The third and fourth points of contact between subunit 𝑖 and its neighbors involve the equivalent interfaces formed with the 𝑖 + 2 and 𝑖 − 2 subunits (Fig. 1L). This interface buries ~400 Å^2^, which is an underestimate given that a nine-residue segment of the BB loop, which forms part of this interface, is missing. In this interface, residues at the start of the TIR domain, βA and αA of the 𝑖 + 2 and 𝑖 − 2 subunits (Q391, Y395, Q406, and L410), interact with residues of subunit 𝑖 in the αE; the BB loop of the 𝑖 + 2 and 𝑖 – 2 subunits (C428, E429, D430, F431, Q432, and V433); αE of subunit 𝑖 (I519, R522, K523, N526, T527, and R532); βD and the αC-βD loop (I487 and D484); and βE (V511 and L513).

### TRIF forms a macromolecular TRIFosome in response to TLR activation

**Fig. 2.**
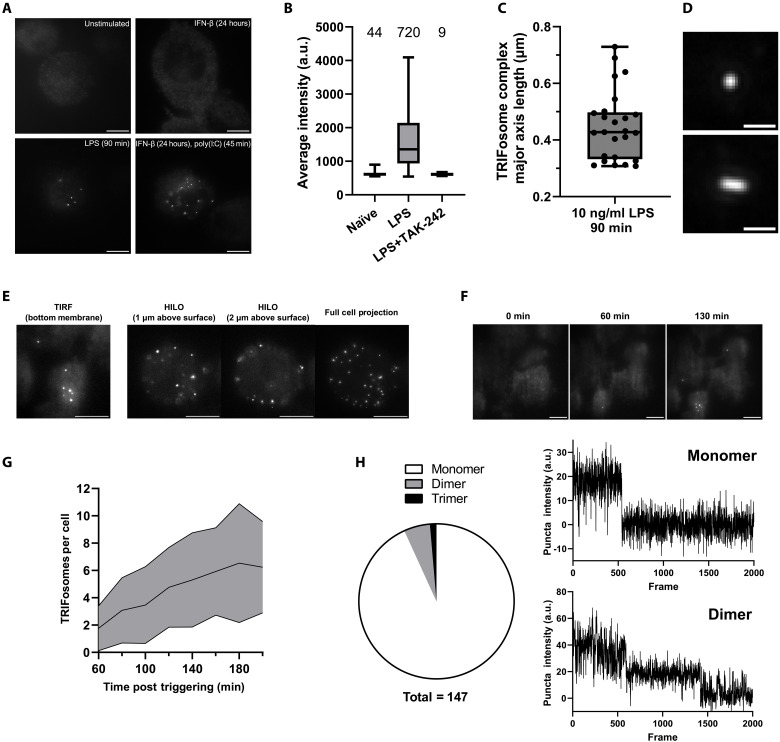
TRIF forms higher-order complexes following TLR activation. (**A**) Representative images of phorbol 12-myristate 13-acetate (PMA)–differentiated THP-1 cells expressing TRIF endogenously tagged with mStayGold. Cells were fixed following TLR4 (LPS, 10 ng/ml) or IFN-β (100 U/ml) priming for 24 hours followed by poly(I:C) (10 μg/ml) stimulation. Scale bars, 10 μm. (**B**) Randomized grid scans of live TRIF-mStayGold cells were taken following different 90-min treatments. Images were analyzed for the presence and intensity of puncta. Box-and-whisker lines represent minimum, lower quartile, median, upper quartile, and maximum values. The values above the plots are the number of puncta detected during analysis. (**C**) The size of TRIFosome complexes range from below the diffraction limit (~300 nm) to above 700 nm post–LPS stimulation. (**D**) Representative images of TRIFosome morphologies show that TRIF can assemble into rod-like structures, extending close to the micrometer range. Scale bars,1 μm. (**E**) Distribution analysis of TRIFosome formation showed that they are present at the membrane (TIRF) and are predominantly found toward the membrane (HILO *z*-stacks). (**F**) Time course of TRIFosome formation in live cells following LPS (10 ng/ml) stimulation shows formation by 60 min with more appearing in the following hour. Scale bars, 10 μm. (**G**) Number of puncta detected per cell following LPS (10 ng/ml) stimulation. TIRF illumination was used to selectively excite fluorophores at the bottom membrane of the cell. The black plot line represents the mean number of puncta; error lines indicate ±SD. *n* = 13. (**H**) Photobleaching step analysis of fixed TRIF-mStayGold in unstimulated, PMA-differentiated THP-1 cells shows that it is predominantly monomeric with occasional higher-order complexes present. Example traces of monomeric and higher-order complexes are shown. a.u., arbitrary unit.

Our structural data support the possibility that TRIF could form a SMOC signaling platform in vivo, but whether this occurs is unknown (*19*, *20*). Using CRISPR-Cas9–mediated homology-directed repair (HDR), we added a fluorescent tag, mStayGold, at the C terminus of the endogenous TRIF gene in THP-1 monocyte-macrophages to visualize TRIF signaling in response to TLR activation. The TRIF-mStayGold cells had equivalent levels of *TRIF* mRNA to those found in WT THP-1 cells and were signaling competent in response to lipopolysaccharide (LPS) stimulation of TLR4, Pam3CysSerLys4 (Pam3CSK4) stimulation of TLR2, and polyinosinic:polycytidylic acid [poly(I:C)] stimulation of TLR3 (fig. S7). To visualize TRIF intracellularly, we used total internal reflection fluorescence (TIRF) and highly inclined and laminated optical sheet (HILO) microscopy of individual proteins in live macrophages over time (Fig. 2 and movie S1). This allows for the observation of individual light diffraction limited TRIF molecules within 200 nm from the coverslip surface (TIRF) and throughout the cell (HILO). We first noted that TRIF was dynamic and distributed widely throughout the cells (movie S2). Endogenous TRIF could only be visualized with maximal laser power, thus requiring the use of mStayGold, which is more photostable than many other fluorescent proteins. This suggests a low level of endogenous TRIF expression. Published data in which TRIF is overexpressed reveal concentration-dependent aggregation, and it is therefore likely that the low endogenous TRIF expression seen in macrophages is an important intracellular regulatory mechanism to prevent spontaneous TRIF oligomerization and hence constitutive TRIF signaling in the absence of TLR activation (*21*). LPS stimulation of TLR4 results in the formation of multiple, mainly spherical macromolecular TRIF complexes (Fig. 2A and movie S3), which were prevented by pretreatment with the TLR4 antagonist TAK-242 (Fig. 2B), confirming that their formation is dependent on receptor stimulation. TRIFosome complexes varied in size from ~200 to 800 nm (Fig. 2, C and D) and were able to fuse (movie S4). TRIF complexes were present intracellularly, likely associated with endosomes, and were also present at the plasma membrane (Fig 2E and movie S5). TRIFosome complexes are established by 60 to 70 min, with a few complexes visible at 50 min after stimulation of TLR4 with LPS (Fig. 2, F and G). To determine whether TRIFosomes were formed in response to TLR3 stimulation, THP-1 cells, which have a low expression of TLR3, were primed with IFN-β for 24 hours, which results in increased expression of both TLR3 and TRIF (fig. S8). Cells were then stimulated with high–molecular weight poly(I:C) to trigger TLR3. TRIFosomes were formed by 45 min in response to TLR3 stimulation (Fig. 2A) and showed enhanced tumor necrosis factor–α (TNFα) production compared to unprimed cells (fig. S8). We determined the approximate stoichiometry of the TRIF-mStayGold signaling complexes by comparing TRIFosome fluorescence intensity to that of monomeric TRIF-mStayGold. Prior to LPS stimulation, TRIF is largely monomeric in the cell, although the occasional small dimeric/trimeric TRIF complexes could be identified (Fig. 2H). In TLR4-stimulated cells, we identified a range of complex sizes, with small complexes containing more than 20 TRIF molecules (fig. S9).

### TRIF signaling is modular over time

**Fig. 3.**
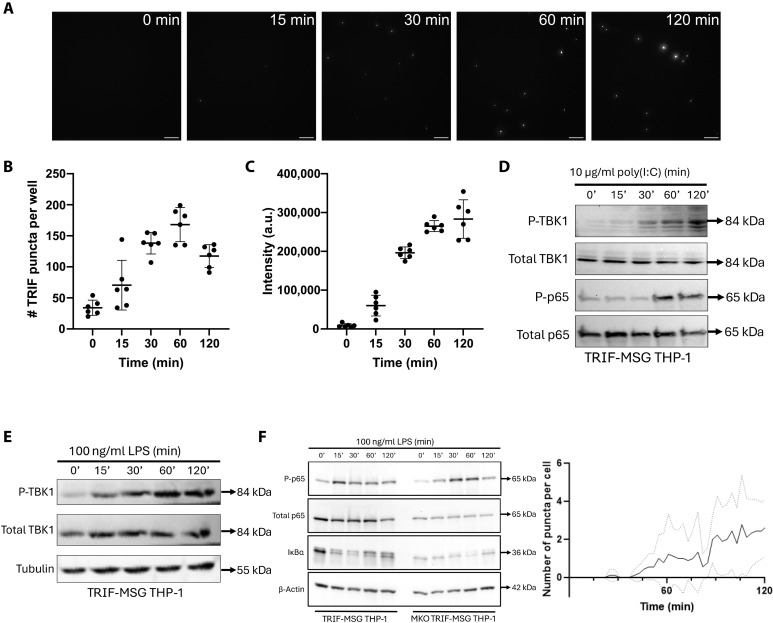
TRIFosomes are active signaling complexes. (**A**) TRIF–mStayGold (MSG) cells were lysed following LPS (100 ng/ml) stimulation over a 120-min time course. Large TRIFosomes were pulled down to the surface by a RIPK1 capture antibody; representative images shown. Scale bars, 10 μm. (**B**) The number of RIPK1-positive TRIFosomes increased to a maximum 60 min following stimulation with LPS (100 ng/ml). (**C**) The mean intensity of RIPK1-positive TRIFosomes increased with time following LPS (100 ng/ml) stimulation. For (B) and (C), data were pooled from three technical replicates each across two biological replicates. Plots indicate mean ± SD; total area imaged per well: 128,000 μm^2^. (**D**) Representative immunoblots showing the kinetics of TBK1 and p65 phosphorylation following poly(I:C) (10 μg/ml) stimulation. (**E**) Immunoblots depicting TBK1 phosphorylation induced by LPS (100 ng/ml) stimulation. (**F**) Left: Representative immunoblots demonstrating delayed phosphorylation of p65 and degradation of IκBα in WT and MyD88-knockout PMA-differentiated TRIF-mStayGold cells after LPS stimulation. Right: Number of puncta detected per cell following LPS (10 ng/ml) stimulation. Measured across nine cells over two biological replicates (solid line, mean; dashed lines, ±SD).

Activation of TRIF by TLR3 or TLR4 activates signaling through Tank binding kinase 1 (TBK1) to trigger interferon regulatory factor 3 (IRF3) and through RIPK1 to activate NF-κB (*22*, *23*). The timing of TRIFosome formation (45 to 60 min) aligns with the well-documented delayed TLR4-TRIF–dependent activation of NF-κB (*24*). TLR4 first activates MyD88-dependent NF-κB signaling, which occurs faster than TLR4-TRIF NF-κB activation. TLR3 only activates TRIF to trigger NF-κB nuclear translocation. We therefore first compared TBK1 and p65 activation after poly(I:C) stimulation of TLR3 in TRIF-mStayGold cells to determine whether TRIFosome formation is functionally linked to NF-κB and IRF3 signaling. TBK1 phosphorylation was detected within 30 min (Fig. 3D) after addition of poly(I:C), before TRIFosome formation, which occurs at ~45 to 60 min. Phosphorylation of the p65 NF-κB subunit was detected at 60 min poststimulation occurring, as expected, shortly after TRIFosome formation (Fig. 3D). LPS-induced activation of TRIF-dependent IRF3 signaling, as measured by TBK1 phosphorylation (Fig. 3E), occurred 15 min after cell stimulation, before TRIFosomes form. We then stimulated TRIF mStayGold cells where MyD88 was deleted by CRISPR-Cas9 gene editing with LPS, as, here, TLR4–NF-κB activation should be dependent only on TRIF. In these experiments, LPS-induced TRIFosome formation and phosphorylation of p65 NF-κB occurred in a similar time frame (Fig. 3F).

These data suggest that while the TRIFosome SMOC forms to activate NF-κB, unexpectedly, TBK1/IRF3 signaling does not require SMOC formation and may even occur from monomeric TRIF protein. To determine the size of the TRIF signaling complex required to associate with downstream signaling proteins, we used single-molecule pull-down (SiMPull) analysis. This technique allows us to visualize the size of the TRIF-mStayGold signaling complex, by using single-molecule fluorescence analysis, that coassociates with signaling proteins. Immunoprecipitation studies, in contrast, only show coassociation of two proteins with no indication of the size of the protein complex required to trigger a signaling pathway. Purified TRIF protein interacts with RIPK1 through its RHIM domain; therefore, an active TRIFosome signaling platform should recruit RIPK1 in the cell (fig. S1). We plated lysates of TRIF-mStayGold THP-1 cells at 0, 0.25, 0.5, 1, and 2 hours poststimulation with LPS onto RIPK1 antibody-functionalized coverslips to pull down TRIFosome complexes. Visualization of the coverslips showed that RIPK1 only associates with the large TRIFosome complexes, from 1 hour after LPS stimulation, confirming that it forms a functional signaling platform (Fig. 3, A to C). These data support a modular mechanism for TRIF-dependent signaling whereby TRIF activation of IRF3 occurs from monomeric or transient lower-order TRIF complexes, but the later TRIF-dependent activation of NF-κB occurs from a large TRIFosome platform. This large TRIFosome could also provide a platform for the diverse outputs of TRIF signaling from inflammatory signaling to the formation of cell death complexes (*25*).

### MyD88, like TRIF, also signals in a modular fashion

**Fig. 4.**
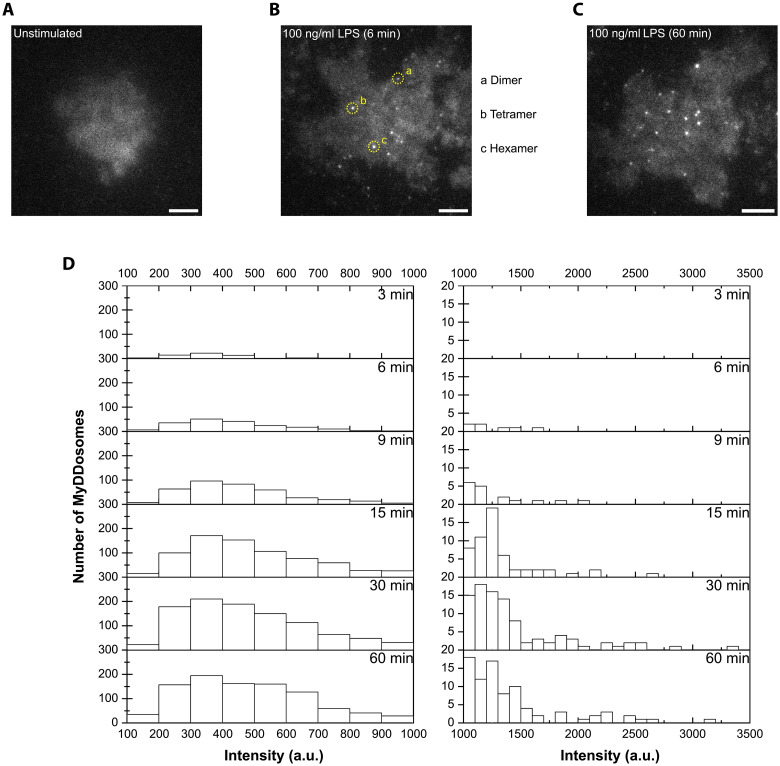
MyD88 forms specific signaling modules. (**A** and **B**) Fluorescence intensity analysis of MyD88-mNeonGreen shows that it is predominantly monomeric in unstimulated cells, but hexamers and larger oligomers appear 6 min after TLR4 activation. The number of MyD88 within a MyDDosome is determined by comparing its fluorescence intensity to that of monomeric MyD88-mNeonGreen. Scale bars, 5 μm. (**C**) Stable MyD88 complexes are still visible 1 hour after TLR4 activation. Scale bar, 5 μm. (**D**) Intensity comparison analysis shows an overall increase in stoichiometry of MyD88 over the course of an hour (66 cells were imaged in four replicates).

We next explored whether modular adaptor complex formation is common to other TLR signaling pathways by investigating MyD88 signaling in response to LPS activation of TLR4. Our earlier structural analysis of MyD88 (*26*) proposed that spontaneous oligomers of this protein may form in vitro so we first tested whether this occurred within cells. Immortalized macrophages generated from mice genetically modified to express endogenous MyD88 tagged with mNeonGreen showed that this protein, like TRIF, is dynamic (movie S6) with 73% being monomeric and 27% forming small MyD88 oligomers (fig. S10). These cells have conserved LPS signaling (fig. S10), and within 6 min of TLR4 activation, MyD88 formed hexameric MyDDosomes (Fig. 4, A and B), as we had seen in our previously published work (*26*, *27*). Rapid MyDDosome formation drives NF-κB translocation to the nucleus, which occurs within 20 min of LPS stimulation (*23*). One hour post–LPS stimulation, stable MyDDosome complexes were still visible in the cells (Fig. 4C) with some MyDDosomes fusing to form super MyDDosomes (Fig. 4D and movie S7) consistent with earlier studies from ourselves [using MyD88-mGFP reconstituted MyD88^−/−^ iBMDMs; (*27*)] and others (*28*). The presence of stable MyDDosome platforms 1 hour after TLR activation is in agreement with recent data, which suggest that these complexes can remain in the cell for many hours as a platform to recruit multiple signaling proteins (*29*). Together, our data therefore support a common modular signaling model for TLR adaptor proteins.

## DISCUSSION

TLR3 and TLR4 signaling uses the large multidomain adaptor TRIF, which, with the RIPK1/RIPK3 kinases, is predicted to form oligomeric TRIFosomes. Here, we show that TRIF forms filaments by homo-oligomerization of both its TIR and RHIM motifs and associates with RIP1/3 kinases through its RHIM domain in vitro, supporting the potential for TRIFosome formation. We show that an oligomeric TRIFosome SMOC forms in macrophages only after TLR3 or TLR4 stimulation to activate NF-κB signaling. Formation of the TRIFosome SMOC is not required, however, for TLR-TRIF–induced TBK1/IRF3 signaling, which occurs from monomeric or transiently lower-order TRIF signaling complexes. We have therefore identified a previously unidentified, SMOC-independent mechanism of TLR signaling.

Our in vivo fluorescence data show that endogenous TRIF is predominantly monomeric, which eventually self-associates into large TRIFosome oligomers in response to TLR3/TLR4 stimulation, suggesting that the filaments we see represent activated TRIF. The NTD and amyloid fibrils formed by the C-terminal RHIM motif are absent in the cryo-EM map. The intervening sequences between the NTD/TIR and the TIR/RHIM are devoid of ordered secondary structure in the AlphaFold model, suggesting that these interdomain regions are likely flexible. On the basis of yeast two-hybrid screening, an interaction between the TIR and NTD domains was inferred (*30*). Its absence in our structure implies that any NTD-TIR association is transient, or that it captures an “activated” form of TRIF. The orientations of the NTD or amyloid fibrils relative to the core TIR filament are unknown, but the structure provides clues regarding possible modes of association of the RHIM motifs. The first involves interaction of the RHIMs of exclusively even (2𝑖 ∈ ℤ) or odd (𝑖 ∈ 2ℤ + 1) numbered filaments, which would result in fibrils whose long axis is aligned with the helical axis of the TIR domain filaments. Alternatively, RHIM oligomerization could involve a zig-zag (…, 𝑖 − 2, 𝑖 − 1, 𝑖, 𝑖 + 1, …) association in which fibrils essentially trace the circumference of the TIR filaments (fig. S2D). The C-terminal helix (αE) of the TIR domain is predicted with high confidence by AlphaFold to span 70 residues, of which only 34 are observed in the cryo-EM map, suggesting that intersubunit interactions in the TIR filament stabilize the proximal half of αE while the rest is highly mobile, influencing the orientation adopted by the self-association of the RHIM motif. During review of this manuscript, a separate study by Manik *et al.* (*31*) has reported a structure for TRIF filaments formed in vitro by a truncated form that lacks 167 residues from the C terminus, which includes the RHIM. As in our full-length structure, the N-terminal tetratricopeptide repeat is not observed and the TIR domain forms a similar helical, two-stranded filament. In the structure of Manik *et al.* (*31*), the TIR domain ends at A535, which is 20 residues shorter than that observed with the full-length protein, implying that the C terminus—presumably via the RHIM—has a stabilizing effect on αE. Our structure also explains the failure of BB loop mutations to abolish TIR-TIR association (fig. S4B). While each protofilament comprises a simple isodesmic assembly, which, on its own, is devoid of cooperative interactions, the assembly of two interacting TIR protofilaments is enhanced by lateral interfilament contacts, which promote cooperativity (*32*). Mutations in the BB loop can be tolerated given its flexibility and, perhaps more importantly, because it is not the only interface responsible for TRIF-TIR self-association. Consequently, oligomeric forms of TRIF are only abolished in the absence of the TIR domain and a nonfunctional RHIM motif as seen with the TRIS-R4A variant. The assembly of the TIR filaments of TRIF suggests that the BB loop of TLR3 interacts directly with the groove formed by the βD/βE strands, an interface, which was also observed in the MAL-TIR filament (*18*). The engagement of the BB loop of TLR3 is envisaged to occur with the 𝑖 + 2 and 𝑖 + 3 subunits of TRIF-TIR for example, which implies that the receptor TIR domains should also be related by a twofold axis.

Our data show that TLR3 or TLR4 activation induces macromolecular TRIFosomes of varying stoichiometries within macrophages. Our data support the concept that both TRIF and MyD88, over time, oligomerize to form large, stable signaling complexes leading to sustained, coordinated, and diverse signaling. Why these larger signaling complexes form and how this leads to more diverse signaling are unclear. Perhaps large signaling platforms provide a distinct compartment to cluster the signaling proteins together to either coordinate simultaneous signaling through different pathways or to deliver sustained signaling to produce a maximal response against pathogen infection. We find that TRIF complex size regulates the physiological outputs of TLR activation. TRIF-dependent IFN signaling occurs while TRIF is predominantly monomeric, but NF-κB activation occurs when macromolecular TRIFosomes are present. The identification of a TLR-activated signaling pathway that occurs through either monomers or transient lower-order complexes (dimer or trimers) is both interesting and unexpected. Prior to our data presented here, TLR signaling was thought to require oligomeric SMOC formation, but our study suggests that this is not the case for TRIF-driven IFN signaling. This suggests diverse ways in which TRIF can trigger signaling.

Our study establishes the importance of TRIFosome assembly for innate immune signal transduction and raises several novel hypotheses to be addressed. First, how do the accessory and downstream factors that signal through TRIF become activated by TRIFosome assembly? A key objective will be to determine the structure of the autoinhibited, monomeric form of TRIF and study how nucleation signals cause activation. A second critical question is to understand how the relative propensities of the TIR and RHIM domain to form filaments cooperate during the formation of the supramolecular TRIFosome. Another key question is how RIPK1 and RIPK3 and other coupling factors such as TNF receptor associated factor 6 (TRAF6) and TBK1 (Fig. 1A) are recognized and activated by TRIF. TRIF, in addition to activating IRF3 and NF-κB, is an important regulator of cell death through RIPK3, raising a further question as to whether both the size and constituents of the TRIF-associated signaling complexes determine the spectrum of cell responses when this signaling protein is activated. Only by determining both the size and protein composition of the TRIF signaling complex coupled to the diverse cellular responses will we be able to determine whether this hypothesis is correct.

Collectively, our data support a concept whereby TLR-driven signaling occurs in a modular fashion with the rapid formation of previously unknown SMOC-independent antipathogen signaling complexes followed by progression to stable SMOC signalling platforms. Determining the structure-function consequences of these diverse signaling responses offers the exciting possibility that could allow uncoupling of protective antipathogen responses from severe inflammatory responses to generate therapeutic targets for chronic inflammatory disease.

## MATERIALS AND METHODS

### Protein expression, purification, and in vitro binding assays

Proteins used in the study are human TRIF (also TICAM-1; UniProt accession Q8IUC6), human RIP1 (UniProt accession Q13546), and human RIP3 (UniProt accession Q9QZL0). TRIF/streptomycin (residues 1 to 712), RIP1-RHIM (residues 496 to 583), RIP3-RHIM (residues 388 to 518) along with all truncations or mutations were cloned into the pMCSG9 expression vector (*33*). The constructs contained an N-terminal hexahistidine tag followed by a maltose binding protein and tobacco etch virus (TEV) protease cleavage site. The integrity of all constructs was confirmed by DNA sequencing. The mutants TRIF-R4A, TRIF-P434H, TRIS-R4A (residues 1 to 217/658 to 712), TRIF-R4A-P434H (residues 153 to 712), TRIFNTD-L49D, -K50D, -L51D, and RIP1-I539D were all generated using the QuikChange II sitedirected mutagenesis kit (Stratagene). All proteins were expressed in *Escherichia coli* BL-21 (DE3) (Novagen) and cultured at 20°C overnight after induction with 1 mM isopropyl-β-d-thiogalactopyranoside at 200 rpm. Cells were harvested; resuspended in 50 mM tris, 500 mM NaCl, 10% glycerol, 0.1% NP-40, and 2 mM β-mercaptoethanol (pH 8) containing one EDTA-free protease inhibitor tablet (Roche); and lysed using an EmulsiFlex (Avestin) homogenizer. The lysate was clarified by centrifugation (35,000g, 40 min) and applied to a 5-ml HiTrap chelating column (GE HealthCare) charged with nickel. The column was washed with 15 to 20 column volumes of 20 mM tris, 150 mM NaCl, and 10% glycerol (pH 8), after which gradient elution was performed with a solution of the same composition but containing 500 mM imidazole. TEV protease was added to pooled protein fractions at 4°C to facilitate tag removal, after which samples were further purified by size exclusion chromatography on a Superdex 200 HiLoad 26/60 (GE) with 20 mM tris, 150 mM NaCl, and 2 mM dithiothreitol (DTT; pH 7.5). Protein concentrations were determined using the estimated molar absorptivity value at 280 nm. Recombinant proteins fused to a streptomycin tag were complexed with proteins in a 1:1 molar ratio in a solution containing 20 mM tris, 150 mM NaCl, 2 mM DTT, and 1 mM EDTA (pH 7.5) and loaded onto a 5-ml StrepTrap HP column (GE) using a peristaltic pump and a flow rate of 0.5 ml/min. Complexes were eluted using two column volumes of the same solution containing 2.5 mM desthiobiotin (Sigma-Aldrich).

### Analytical ultracentrifugation and circular dichroism

Sedimentation velocity measurements were performed using a Beckman XL-A centrifuge at 20°C and a sample concentration of 4 μM in 50 mM tris, 50 mM NaCl, and 0.3 mM Tris(2-carboxyethyl) phosphine hydrochloride (TCEP) (pH 8). Both sample and reference volumes were 400 μl, and data were acquired at 45,000 rpm using an An60Ti rotor (Beckman Coulter). Buffer viscosity and density were estimated using SEDNTERP (*34*), and the data were analyzed with SEDFIT (*35*). Circular dichroism spectra in the far-ultraviolet were recorded on an Aviv 410 spectropolarimeter. The instrument bandwidth, scan increment, and averaging time were 1 nm, 0.5 nm, and 1 s, respectively. Data were acquired at 20°C using a cell of pathlength 0.1 cm. Five spectra were accumulated and averaged, and the resulting spectrum was corrected by subtracting a buffer only spectrum.

### Electron microscopy and model building

A 4-μl sample of TRIF (5 μM) was applied to a QUANTIFOIL R1.2/1.3 holey carbon grid, which had been glow-discharged twice, 1 min on each side, at 25 mA using a PELCO easiGLOW (Ted Pella Inc.) instrument. Grids were blotted using 55/20-mm diameter paper (Agar Scientific) and frozen in liquid ethane using a Vitrobot Mark IV (FEI) at 100% humidity, 277 K, and a blot force of +10. Grids were imaged using a Titan Krios (FEI) operating at 300 kV with a K3 (Gatan) direct electron detector. Movies were recorded using a nominal magnification of 81,000 ± 1620 and a calibrated pixel size of 1.066 Å. Forty-six frames were acquired with a per-frame dose of 1.15 e/Å^2^ and defocus range of 0.1 to −2.6 μm. Estimates of the helical symmetry were calculated from the averaged power spectrum of aligned patches obtained from filaments (100 micrographs) that were windowed using a box size of 200 pixels using *sxhelixboxer* from the SPARX (*36*) package. This gave an axial rise and azimuthal rotation per subunit of 16 Å and 180°, respectively. Subsequent calculations were performed using CryoSPARC (*13*). Movie stacks were motion-corrected with Patch Motion Correction and contrast transfer function (CTF)–estimated with Patch CTF Estimation (fig. S5). Filament Tracer was then used to pick filaments using a filament diameter of 62 Å. The separation between segments was 0.25 times the filament diameter, and the minimum and maximum diameters for template free tracing were 58 and 66 Å, respectively. This produced 8 million overlapping segments, which were extracted (box-size, 216 pixels) and subjected to reference-free two-dimensional (2D) classification using 80 classes, a batch-size per class of 300 and 30 online-EM iterations. Two classes containing ~740,000 particles were selected, and a second round of reference-free 2D classification was done, which yielded 500,264 particles. Ab initio reconstruction using an initial mini-batch size of 300 and a maximum resolution of 6 Å was used to produce a volume, which was then refined. Estimates of the helical symmetry obtained from indexing the averaged power spectrum were used to bootstrap helical refinement. The maximum out-of-plane tilt was 5°, and the resolution to begin real-space symmetrization was set to 5 Å. The refined helical symmetry values obtained were 16.1 Å and 178.3° for the rise and twist, respectively. A resolution of 3.5 Å was obtained using the Fourier shell correlation at 0.143 between independently refined half-maps. However, this is an overestimate as the d_99_ value (*37*) is 4.2 Å. MOLREP (*15*) was used to place six copies of an AlphaFold (*14*) model of the TIR domain of TRIF followed by iterative real-space refinement and model building using PHENIX (*16*) and COOT (*17*), respectively. TRIF and TRIF-R4A were negatively stained with uranyl acetate and imaged with a Tecnai G2 (FEI) electron microscope operated at 200 kV and equipped with an XR30B camera (AMT). Samples of TRIF for treatment with the protease subtilisin were prepared in 100 mM tris, 8 M urea, and 5 mM TCEP (pH 8.5), vortexed, and incubated at room temperature for 30 min. Iodoacetamide (10 mM) was then added, and the solution was left in the dark for 30 min. The reaction was diluted to 4.8 M urea with 100 mM tris (pH 8.5), and subtilisin (Sigma-Aldrich) was added at an enzyme-to-substrate ratio of 1:50 and incubated at 37°C for 2 to 3 hours, after which methanoic acid (5%, v/v) was added.

### Cell culture and stimulation

THP-1 cells were cultured in suspension in RPMI-1640 complete medium (RPMI-1640, 10% fetal calf serum, 2 mM l-glutamine, and penicillin-streptomycin), incubated at 37°C and 5% CO_2_. In preparation for imaging, cells were differentiated using 50 nM phorbol 12-myristate 13-acetate (PMA; Promega) in plates or chambers, depending on the microscope being used. After 24 hours, cells were adherent and were incubated with fresh media for 24 hours. In the case of poly(I:C) treatment, cells were stimulated with IFN-β (100 IU/ml; Abcam, ab71475) during this 24-hour step to induce expression of TLR3. For experiments involving inhibition, cells were treated with 1 μM TAK-242 (Merck) for 1 hour prior to addition of TLR agonists.

Bones from a male mouse expressing MyD88-mNeonGreen were a gift from M. Taylor (Max Planck Institute for Infection Biology, Germany), which were subsequently isolated and immortalized to obtain immortalized bone marrow–derived macrophages (iBMMs) by K. Fitzgerald (UMass Chan Medical School, USA). iBMM MyD88-mNeonGreen were cultured in complete Dulbecco’s modified Eagle’s medium [DMEM; 10% fetal bovine serum (FBS), 2 mM l-glutamine, 1 mM sodium pyruvate, penicillin (100 U/ml), and streptomycin (100 μg/ml)] at 37°C and under 5% CO_2_ atmosphere. iBMM MyD88-mNeonGreen were kept growing until passage 8.

### THP-1 TRIF-mStayGold CRISPR-Cas9 gene editing

THP-1 cells were modified to introduce an mStayGold tag (*38*) to the C terminus of TRIF. Program V-001 on a Lonza Nucleofector Transfection 2b Device was used to introduce recombinant Cas9 (IDT), trans-activating CRISPR RNA (IDT), CRISPR RNA (crRNA) targeted to the end of *TICAM1* (designed using ChopChop (*39*); synthesized by IDT), electroporation enhancer (IDT), and an single-stranded DNA HDR template encoding mStayGold to the cells. Cells were placed into RPMI complete medium with HDR Enhancer V2 (IDT) and pan-caspase inhibitor (Invivogen) for 24 hours. Cells were resuspended in fresh RPMI complete medium and allowed to grow until genomic DNA could be extracted and the introduction of the tag in the bulk population is confirmed by polymerase chain reaction (PCR) (Table 1). The crRNA sequence was GGTGGTCAGGCAAGGACACG and the HDR template sequence CACAGCCAGCAGCCTTTCCACAGTCACTGCCCTTCCCGCAGTCCCCAGCCTTCCCTACGGCCTCACCCGCACCCCCTCAGAGCCCAGGGCTGCAACCCCTCATTATCCACCACGCACAGATGGTACAGCTGGGGCTGAACAACCACATGTGGAACCAGAGAGGGTCCCAGGCGCCCGAGGACAAGACGCAGGAGGCAGAAATGGCCTCAACCGGCGAGGAGCTTTTTACTGGAGTAGTTCCCTTTAAATTTCAGTTGAAGGGGACGATTAACGGAAAGTCTTTTACCGTTGAGGGGGAAGGAGAGGGAAATAGCCACGAAGGCTCACACAAGGGAAAGTACGTGTGCACAAGTGGCAAGCTCCCAATGTCTTGGGCAGCGCTTGGAACGAGCTTTGGGTACGGCATGAAGTATTATACCAAATATCCGTCCGGGCTTAAGAACTGGTTCCATGAAGTAATGCCTGAAGGCTTCACTTACGACAGACATATACAGTACAAAGGAGATGGCTCCATTCACGCCAAACATCAACATTTTATGAAAAATGGGACCTACCACAATATCGTAGAGTTCACAGGCCAGGATTTTAAGGAAAACTCCCCGGTACTGACCGGCGACATGGATGTTTCACTGCCAAACGAGGTTCAACACATACCGATAGATGATGGAGTTGAGTGTACCGTGACTCTGCAGTATCCTTTGCTCAGTGATGAAAGCAAATGTGTGGAGGCCTACCAGAACACCATTATCAAGCCTCTTCACAATCAGCCTGCTCCCGACGTGCCTTTCCATTGGATCAGAAAGCAATATACTCAATCAAAGGATGACACAGAGGAGCGGGATCACATTATTCAAAGCGAAACACTGGAGGCACACTTGTGACGGCGTGTCCTTGCCTGACCACCTGGGGAACACCCCTGGACCCAGGCATCGGCCAGGACCCCATAGAGCACCCCGGTCTGCCCTGTGCCCTGTGGACAGTGGAAGATGAGGTCATCTGCCACTTTCAGGACATTGTCCGGG-AGCCCTTCATTTAGGACAAAACGGGCGCGATGATGC-CCTGGCTTTCAGGGTGGTCA.

**Table 1.** TRIF-mStayGold sequencing primers.

List of diagnostic sequencing primers		
	Forward primer (5′–3′)	Reverse primer (5′–3′)
External primers	ACTTGTCCTACCAGGCACAGAT	TAAAAGACCGTAGCAATACCCC
Internal primers	TTATTCAAAGCGAAACACTGGA	TACTTTCCCTTGTGTGAGCCTT

Preliminary microscopy analysis showed the presence of cells with a weak fluorescent signal, but these could not be separated by fluorescence-activated cell sorting. Instead, cells were subjected to manual single-cell cloning, and homozygotic TRIF-mStayGold cells were identified by diagnostic PCR followed by Sanger sequencing of the region covered by the HDR template. The isolated single-cell clones were confirmed to be mStayGold-positive by fluorescence microscopy.

### RNA isolation, cDNA preparation, and qPCR

Total RNA was extracted from the homogenized lysates by PureLink mini RNA isolation kit (12183018A), and isolated RNA was converted to cDNA using Quantitect reverse transcription kit (205311). Quantitative PCR (qPCR) was performed for required primers (Table 2) using Bio-Rad thermal cycler CFX384.

**Table 2.** qPCR primers.

List of primers		
Transcript	Forward primer (5′–3′)	Reverse primer (5′–3′)
**GAPDH**	CAACTCCCTCAAGATTGTCAGCA	GGCATGGACTGTGGTCATGA
**TRIF**	AGCGCCTTCGACATTCTAGGT	AGGAGAACCATGGCATGCA
**TRIF-mStayGold**	ACCACGCACAGATGGTACAG	TTTCCCTTGTGTGAGCCTTC
**TLR3**	CCTGGTTTGTTAATTGGATTAACGA	TGAGGTGGAGTGTTGCAAAGG
**TNFα**	CCTCTCTCTAATCAGCCCTCTG	GAGGACCTGGGAGTAGATGAG
**IFN-β**	AAACTCATGAGCAGTCTGCA	AGGAGATCTTCAGTTTCGGAGG

### Western blotting

For immunoblotting experiments, 0.5 million THP-1 cells of the desired genotype (WT THP-1 or TRIF-mStayGold THP-1) were plated in a 48-well plate and differentiated using 50 nM PMA (356150050) for 24 hours. Following differentiation, cells were allowed to recover for 24 hours in PMA-free RPMI medium and then stimulated with either LPS (200 ng/ml) (tlrl-pb5lps) or high–molecular weight poly(I:C) (1 μg/ml) (tlrl-pic). After stimulation, cells were lysed in RIPA buffer (89900) containing a protease and phosphatase inhibitor cocktail (1861280) along with 1× Laemmli buffer. The lysates were heated to 95°C to denature proteins and stored at −20°C until further use. Prepared lysates were loaded onto an 8% SDS–polyacrylamide gel electrophoresis gel and electrophoresed for 180 min. Proteins were subsequently transferred to a polyvinylidene difluoride membrane over 2 hours at 380 mA. Following transfer, the blots were blocked with 5% milk (for nonphosphorylated antibodies) or 5% bovine serum albumin (for phosphospecific antibodies) for 1 hour at room temperature. The membranes were then incubated with the respective primary antibody for 2 hours at room temperature (Table 3). After three washes with 1% TBST (tris-buffered saline + Tween 20), the blots were incubated with the appropriate secondary antibody for 1 hour at room temperature (Table 3). Final washes with TBST were performed before developing the blots using a chemiluminescence method with ECL solution (RPN2236) on the iBright 1500 imaging system.

**Table 3.** Western blot antibodies.

Antibody	Company	Catalog number	Dilution used for IB
P-p65	CST	93H1	1:1000
Total p65	CST	D14E12	1:1000
p-IRF3	CST	4D4G	1:1000
Total IRF3	CST	D83B9	1:1000
p-TBK1	CST	D52C2	1:1000
Total TBK1	CST	3013S	1:1000
IκBα	Santa Cruz	sc-371	1:1000
α-tubulin	Sigma-Aldrich	3013S	1:5000
HRP anti-rabbit	CST	7074S	1:5000
HRP anti-mouse	CST	7076S	1:5000

### Cytokine quantification

Secreted cytokines were measured using enzyme-linked immunosorbent assay (ELISA) from appropriately diluted experimental supernatants in growth media. All assays were performed according to the manufacturers’ instructions. TNFα levels were quantified using the Human TNFα DuoSet ELISA (DY210-05, R&D Systems), and IFN-β levels were measured using the VeriKine-HS Human Interferon Beta Serum ELISA Kit (41415-1, PBL Assay Science).

### Microscopy

Live and fixed cell imaging was carried out on two custom-built TIRF microscopes. Both microscopes were fitted with incubators to maintain temperature at 37°C for long-term imaging. For microscope A, lasers with wavelengths at 488 nm (iBeam smart, Toptica) and 561 nm (Cobolt Jive, HÜBNER) were combined into a common beam path using suitable dichroic mirrors. Flat field illumination was used and was achieved by coupling the combined beam coupled into a square-core optical fiber (05806-1 Rev. A, CeramOptec) and using a vibration motor (304-111, Precision Microdrives) fixed onto the fiber to remove speckles (*40*). The beam was focused onto the back focal plane of the objective lens [100× Plan Apochromat TIRF, numerical aperture (NA) 1.49, oil immersion, Nikon Corporation], and the fluorescence emission was collected by the same objective. The emission was filtered by a quad band emission filter to remove the excitation light and then further cleaned by suitable emission filters (488: FF03-525/50-25, 561: FF01-600/37-25, Semrock). Fluorescence was recorded using an sCMOS camera (Prime BSI Express, Teledyne Photometrics). The objective was mounted onto an inverted optical microscope (Ti-E Eclipse, Nikon Corporation), and the Nikon perfect-focus system was used to maintain focus throughout image acquisition. The image acquisition was controlled through Micro-Manager 1.4, while laser powers were controlled through the use of the manufacturer’s software, or by attenuation using neutral density filters. Exposure times and laser powers were set for each experiment such as to minimize any unwanted photobleaching, while maximizing the acquired fluorescence signal. The TIRF angle was controlled based on the experimental requirements to allow for either TIRF imaging of complexes on the basal cell surface, or HILO imaging for complexes off the bottom surface. Microscope B used a similar setup with a few modifications. The 488-mm laser line (iBeam smart, Toptica) was expanded using Galilean beam expanders and focused to the back focal plane of the objective using Köhler lens to generate a Gaussian illumination profile. The 100× objective was mounted on an inverted optical microscope (Ti2 Eclipse, Nikon Corporation). Fluorescence emission was cleaned by suitable emission filters (FF01-520/44-25 + BLP01-488R, Semrock), and fluorescence was recorded using an electron-multiplying charge-coupled device with an EM-gain of 250 ADU/photon (Evolve 512 Delta EMCCD, Teledyne Photometrics). The image acquisition was controlled through Micro-Manager 2.0. TRIFosome count and intensity were measured using native Fiji commands as follows: setThreshold(500, 1000000, “raw”); run(“Convert to Mask”); run(“Watershed”); run(“Analyze Particles...”, “size=5-Infinity pixel show=Overlay display clear summarize add”). Photobleaching step analysis was conducted using QuickPBSA (*41*).

### MyDDosome live-cell imaging

iBMM MyD88-mNeonGreen were placed onto a glass-bottom confocal dish (ibidi GmbH, catalog no. 81158) and left to adhere overnight. The complete DMEM was then replaced by FluoroBrite complete DMEM [FluoroBrite DMEM, 10% FBS, 2 mM l-glutamine, 1 mM sodium pyruvate, penicillin (100 U/ml), and streptomycin (100 μg/ml)] containing 25 mM Hepes. iBMM MyD88-mNeonGreen were stimulated by adding LPS (100 ng/ml) and were imaged in TIRF. The focus plane was set to the bottom surface of cells, and one frame was captured in the same field of view (FOV) every 3 min. Image analysis was performed using Fiji (National Institutes of Health) with a custom automation macro. All images were first background-subtracted by Gaussian blurring (radius = 4 pixels). MyDDosome puncta were identified using ThunderSTORM (*42*), and their intensity was subsequently measured. Puncta were distinguished from noise by having threefold intensity significance compared to the background.

### MyD88-mNeonGreen photobleaching

One-milliliter iBMM MyD88-mNeonGreen suspension (cell concentration = 10^7^ cells/ml) was centrifuged at 4°C, 400*g* for 5 min to collect cell pellets. The pellets were washed twice with 3 ml of ice-cold PBS and then resuspended in 500 μl of lysis buffer (50 mM tris-HCl, 150 mM NaCl, and 1% NP-40, pH 7.4) containing protease inhibitors (cOmplete Roche, catalog no. 4693116001) and phosphatase inhibitors (PhosSTOP Roche, catalog no. 4906845001). The mixture was incubated on ice for 30 min and centrifuged at 4°C, 16,000 rpm for 30 min to remove cell debris. The supernatant (200 μl) was transferred into a glass-bottom eight-well chamber (ibidi GmbH, catalog no. 80807) and imaged in TIRF. A total of 1000 frames were continuously captured at the same FOV, with an exposure time of 100 ms to achieve photobleaching. Monomeric MyD88-mNeonGreen proteins were identified by a single intensity drop in the photobleaching intensity curves, and their intensity was determined using the same method as in MyDDosome live-cell imaging.

### THP-1 TRIF-mStayGold cell lysate pull-down and imaging

THP-1–mStayGold cells were differentiated in a 48-well multiwell plate (CELLSTAR, Greiner) with a seeding density of 300,000 cells per well (same differentiation protocol as above). The cell medium was replaced with either fresh complete RPMI for the naive condition or medium supplemented with LPS (100 ng/ml) for the required duration (LPS Ultrapure, Invivogen). Following stimulation, cells were lysed with ice-cold lysis buffer (50 mM tris-HCl, pH 7.5), 150 mM NaCl, 5 mM MgCl_2_, and 1% (v/v) NP-40, supplemented with Halt protease and phosphatase inhibitor cocktail (1861280).

The SiMPull measurements were conducted with minor modifications based on previous protocols (*43*). Coverslips were initially treated with a Rain-X coating solution (Rain-X mixed with isopropanol at a 1:1 ratio and filtered twice using a 0.2-μm filter, Millex, SLGV004SL) for 10 min, followed by curing for 90 min. The coated surface was then incubated with an F-127 coating solution (10 mg/ml; composed of 1% biotinylated F-127 and 99% nonbiotinylated F-127 in PBS) to achieve selective passivation. The passivated coverslip was subsequently treated with NeutrAvidin in PBS (0.2 mg/ml; Thermo Fisher Scientific, 31000) for 20 min and then with biotinylated goat anti-rabbit immunoglobulin G (IgG) (1.6 μg/ml; Abcam, ab6720) for 30 min, with a 3× PBST (PBS + 0.05% Tween 20) wash performed between these two steps. Either an RIP rabbit monoclonal antibody (D94C12, no. 3493, Cell Signaling) or a rabbit IgG isotype control (Abcam, EPR25A) was then introduced to independent imaging wells at a concentration of 1.6 μg/ml for 30 min, followed by 3× washes with PBST. The samples were added to the functionalized imaging wells and incubated for 5 hours at room temperature. Following this incubation, the coverslip was imaged directly without any additional washing.

### Imaging TRIFosomes with a light sheet microscope

TRIFosome localization in the full volume of THP-1–mStayGold cells was cross-validated by imaging with a bespoke open-top single-objective light sheet microscope based on the oblique plane microscopy configuration (*44*). The instrument’s design was similar to that described by Yang *et al.* (*45*). The core imaging component consisted of a primary 60× NA 1.27 water-immersion objective (CFI Plan Apochromat IR 60XC WI, Nikon) mounted on an inverted microscope (Eclipse Ti-U, Nikon) for both light sheet illumination and emission collection. A 488-nm continuous diode laser (200 mW, LBX-488-200, Oxxius) was expanded, collimated, and then passed through cylindrical lens to produce a 1D Gaussian beam. A quad-band dichroic mirror (Di01-R406/488/561/635, Semrock) was used to split the illumination and emission wavelengths, following which, it propagated through two 4f systems before entering the primary objective at one edge of the back focal plane to produce a light sheet inclined at 30°. Volumetric imaging was achieved by rapid scanning of the light sheet using a Galvo mirror (GVS201, Thorlabs), with its operation controlled by a home-written LabView script.

Fluorescence collected by the primary objective was transmitted through the quad-band dichroic mirror and relayed onto the remote focusing model (RFM). RFM consisted of a 40× secondary air objective (CFI Plan Apochromat Lambda D Air, NA 0.95, Nikon) whose pupil plane was conjugated with that of the primary objective, a bespoke glass-tipped tertiary objective (AMS-AGY v1.0, NA 1, Calico lab) oriented at the same 30° angle to the optical axis, a tube lens (effective focal length = 321 mm), and an sCMOS camera (Photometrics Prime 95B).

Volumetric scans of cells were achieved using a home-written automation script in Micro Manager 2.0. During each volumetric scan, the light sheets from the 488-nm (44.4 W/cm^2^) lasers scanned through the sample. The camera was synchronized with the Galvo scanning, having an exposure time of 30 ms. Each volumetric scan, consisting of 100 frames with a full 1200 × 1200 pixel FOV, was acquired in ~4 s. The acquired images from each volumetric scan were deskewed and reconstructed into 3D projections or *z*-stacks using a custom Python script.
