# Supplementary material for: Toll-like receptor signaling outcome is determined by the stoichiometry of the endogenous TRIFosome
Source: Sci Adv. 2026 Mar 6;12(10):eaeb9507. doi: 10.1126/sciadv.aeb9507 (PMC12965317; doi:10.1126/sciadv.aeb9507)
Supplement: Supplementary file 1 — Figs. S1 to S11 Tables S1 and S2 Legends for movies S1 to S7 [file sciadv.aeb9507_sm.pdf]

Science Advances
AAAS

Supplementary Materials for

Toll-like receptor signaling outcome is determined by the stoichiometry of the endogenous TRIFosome

Martin C. Moncrieffe *et al.*

Corresponding author: Martin C. Moncrieffe, mcm35@cam.ac.uk; Clare Bryant, ceb27@cam.ac.uk

Sci. Adv. **12**, eaeb9507 (2026)
DOI: 10.1126/sciadv.aeb9507

The PDF file includes:

Figs. S1 to S11
Tables S1 and S2
Legends for movies S1 to S7

Other Supplementary Material for this manuscript includes the following:

Movies S1 to S7

Extended Data Fig. S1

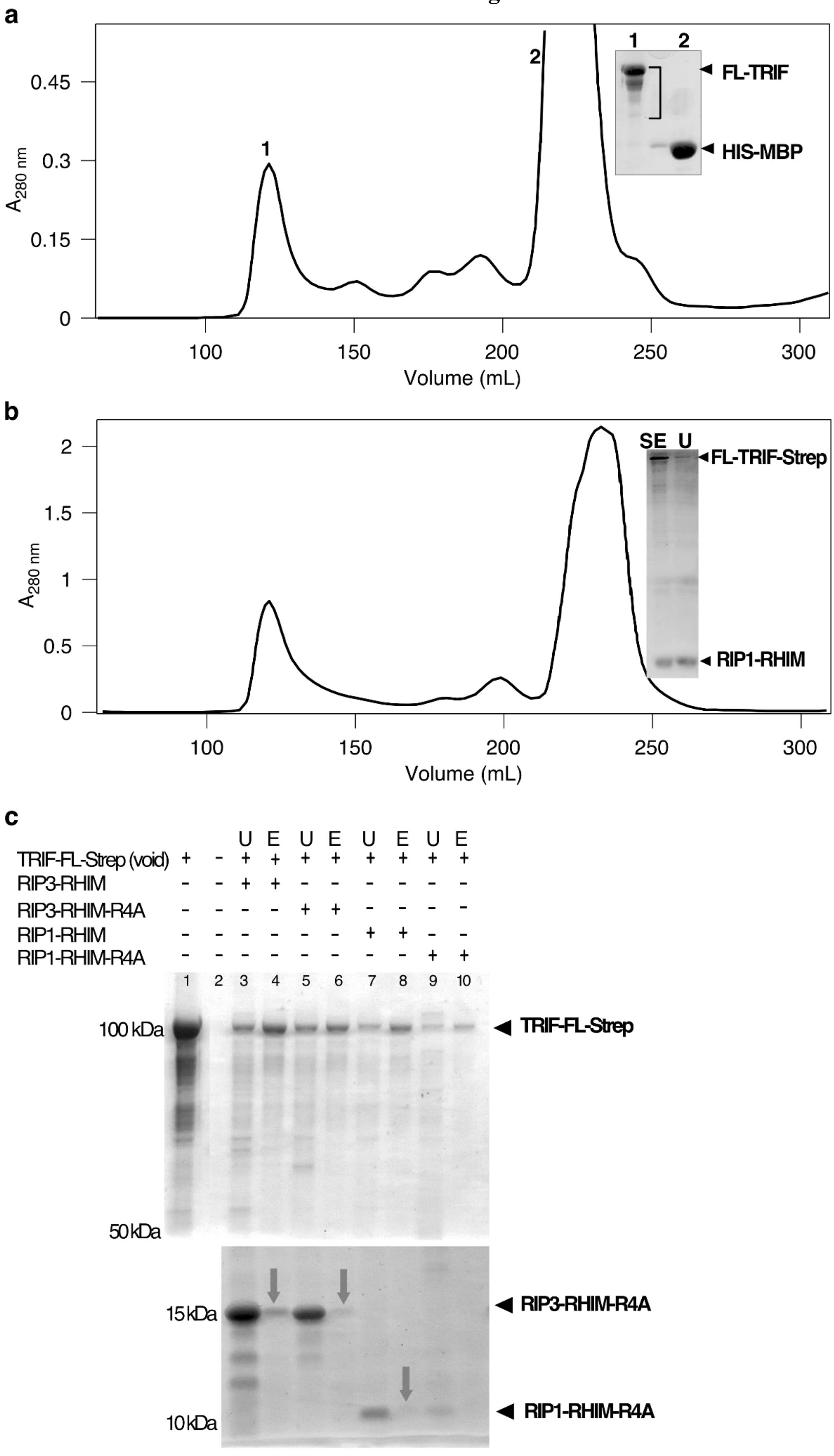

Extended Data Fig. S2

a

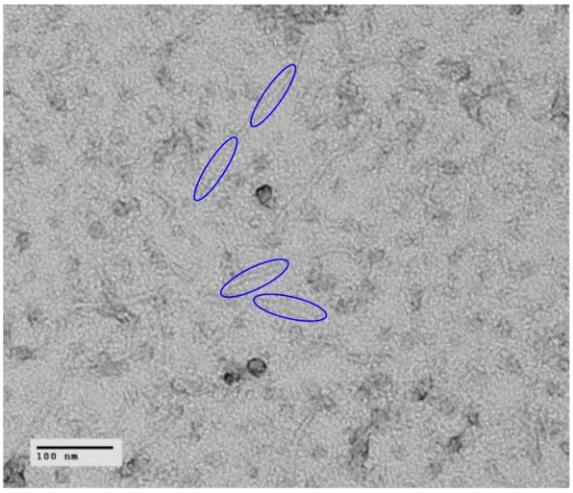

b

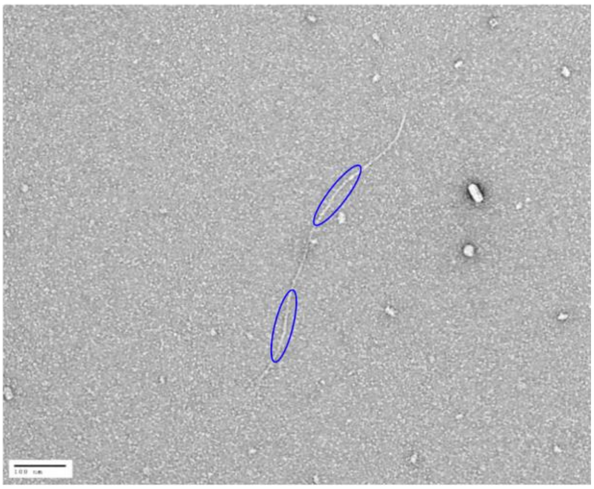

c

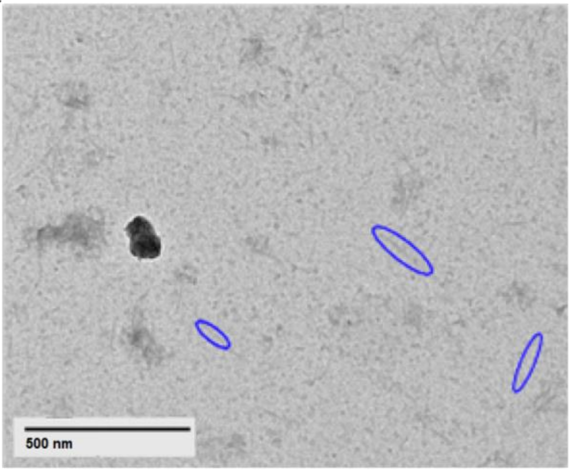

d

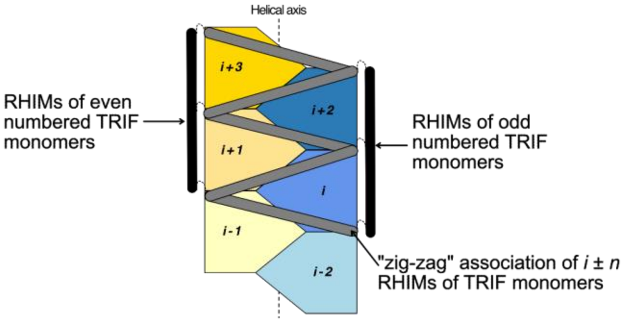

Extended Data Fig. S3

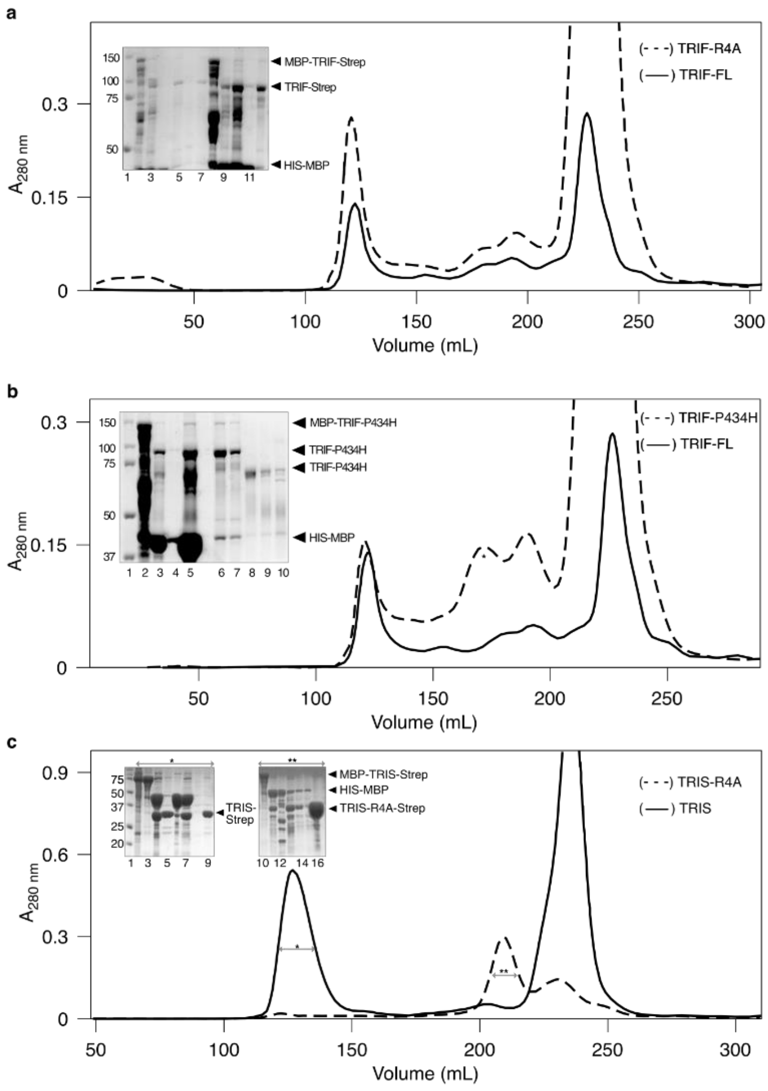

Extended Data Fig. S4

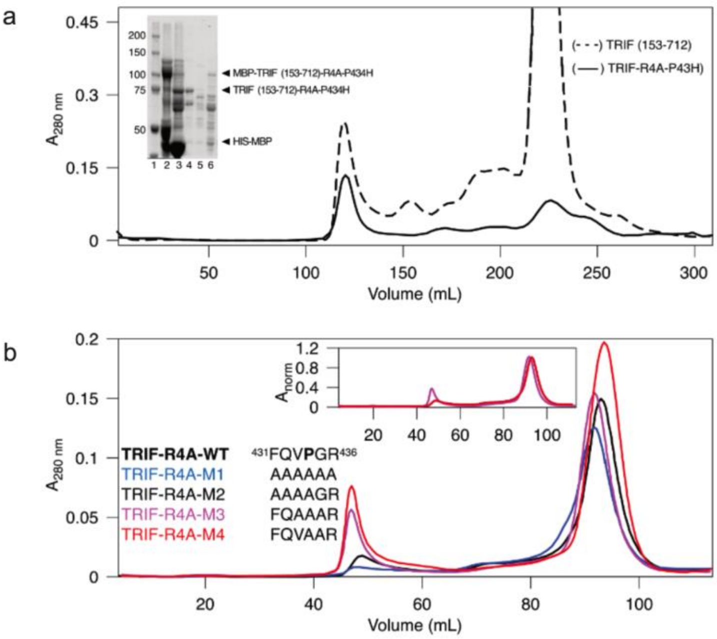

Extended Data Fig. S5

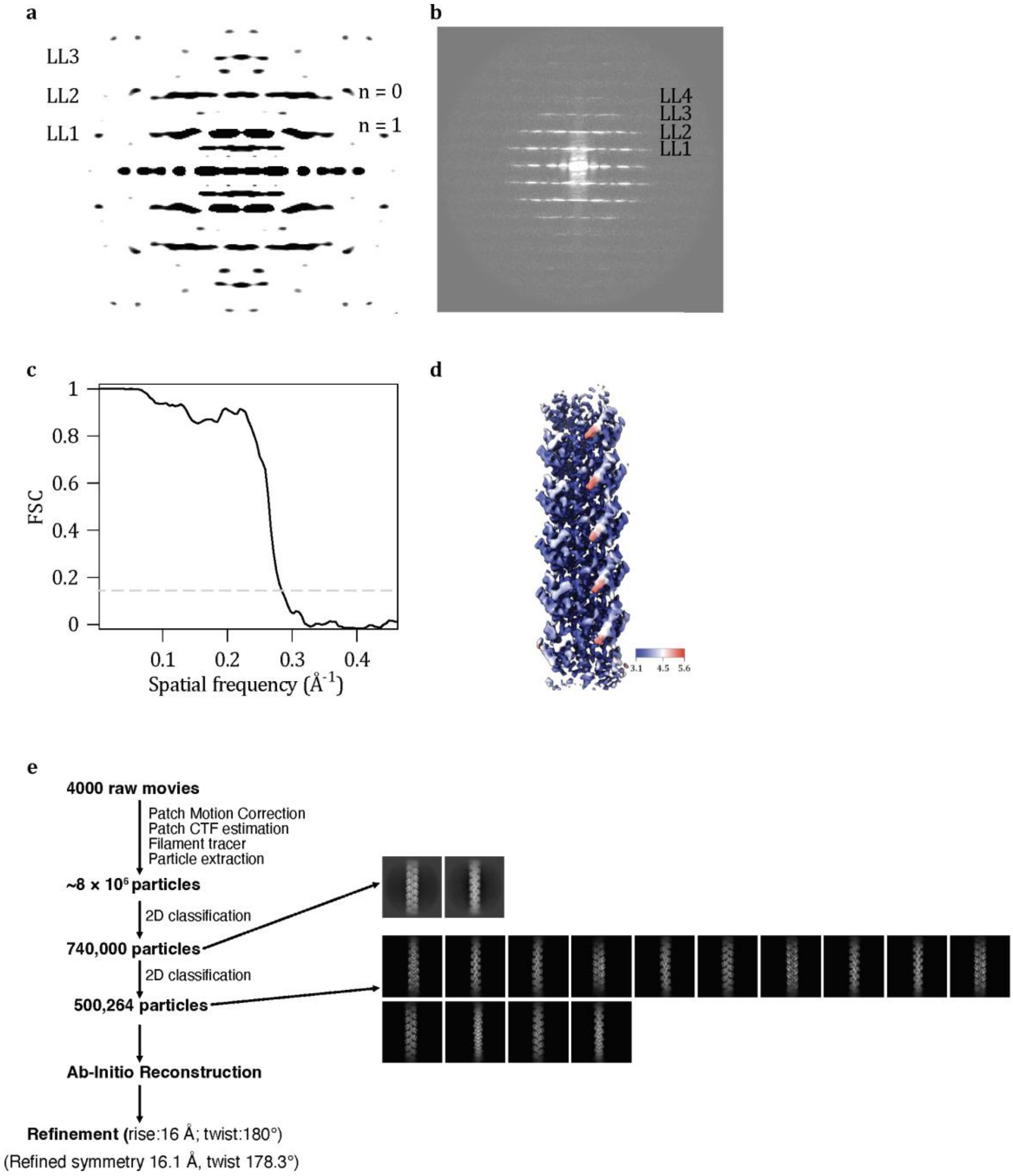

Extended Data Fig. S6

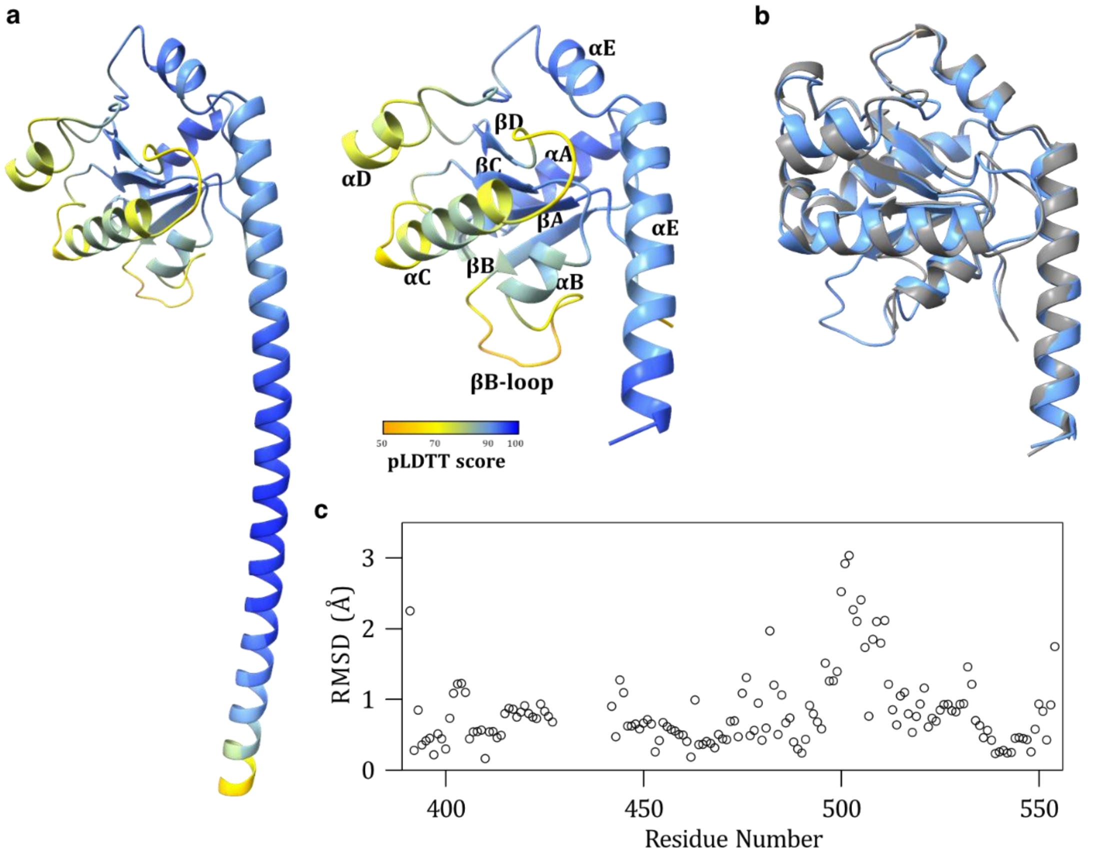

Extended Data Fig. S7

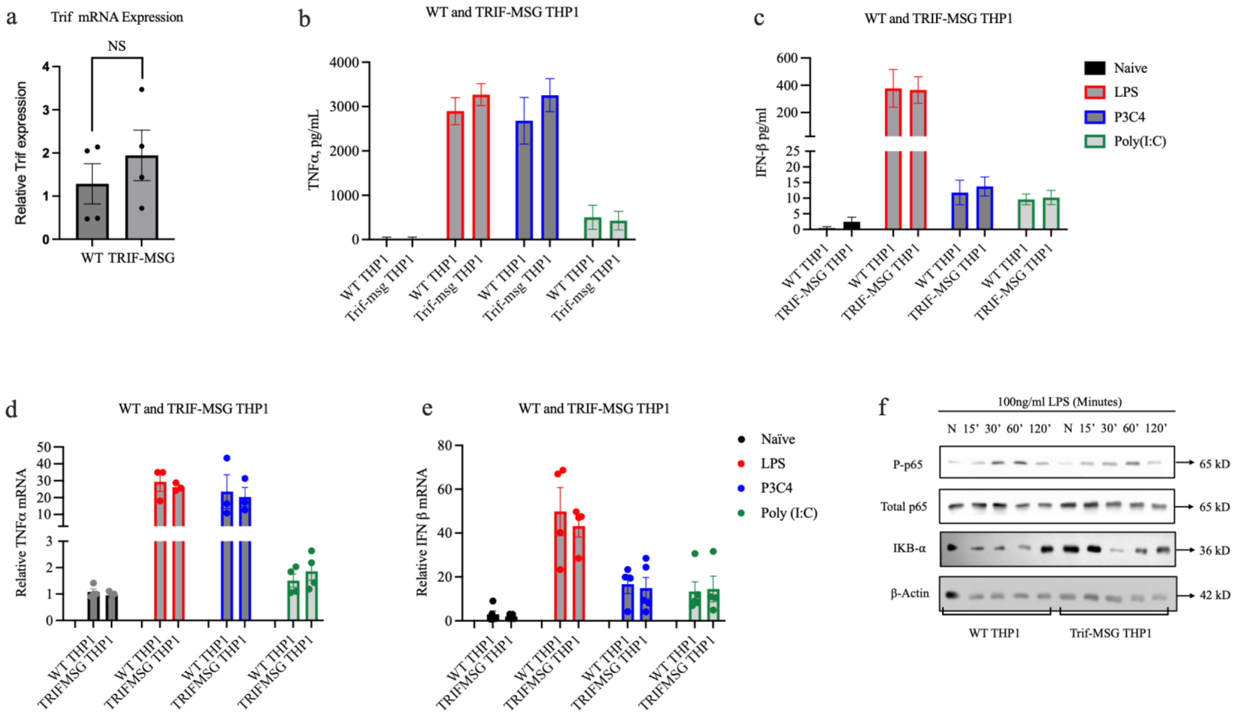

Extended Data Fig. S8

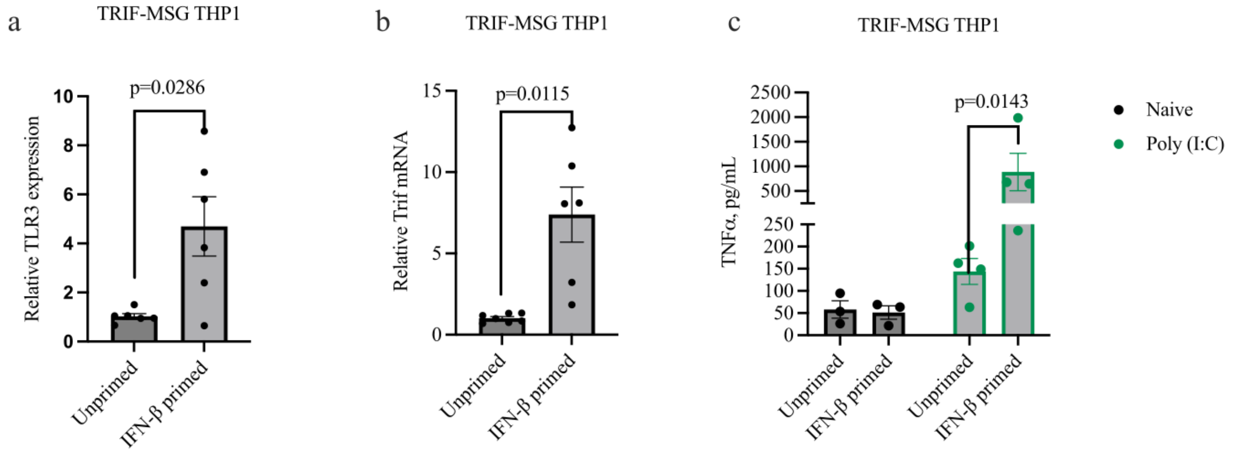

Extended Data Fig. S9

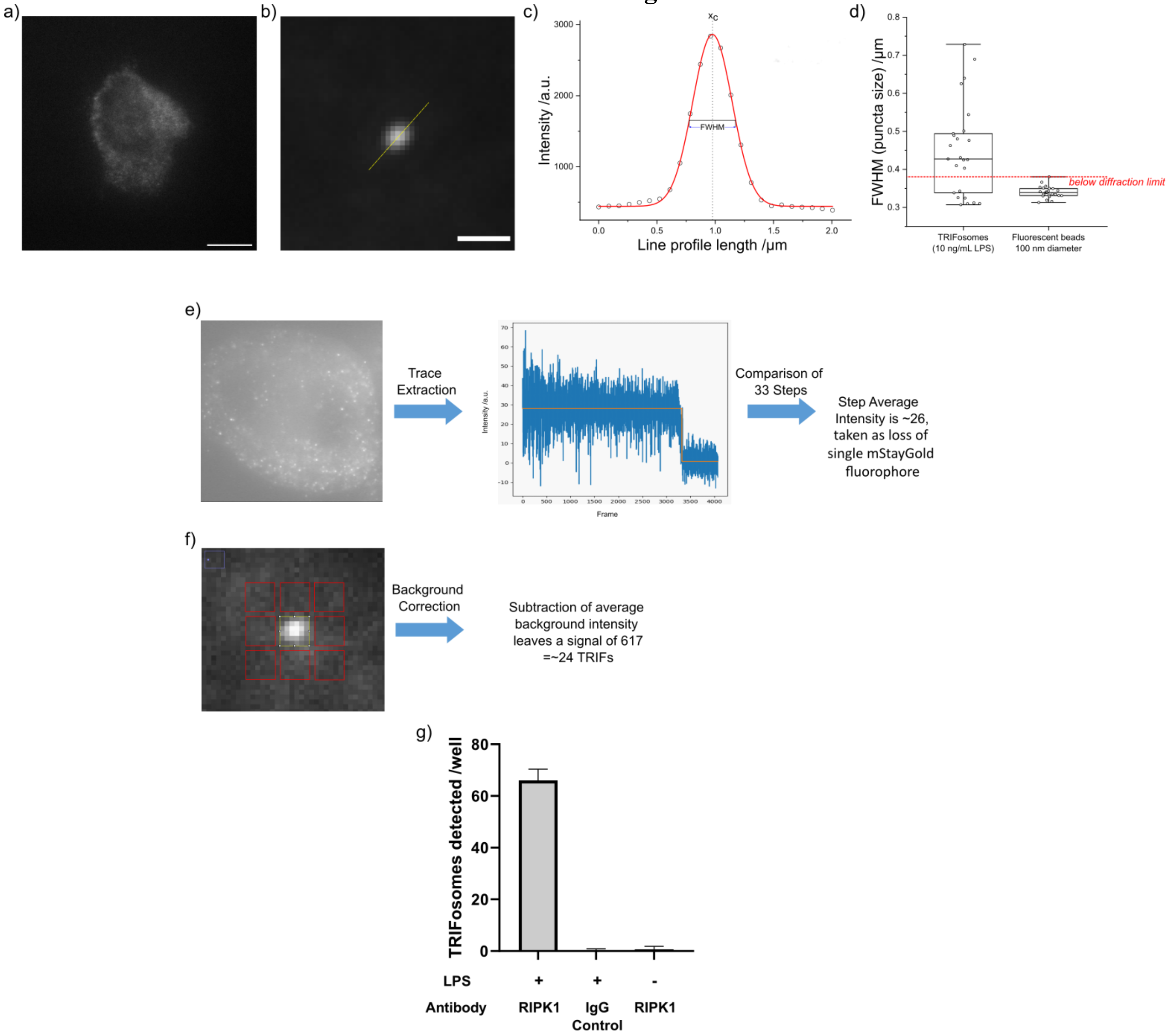

Extended Data Fig. S10

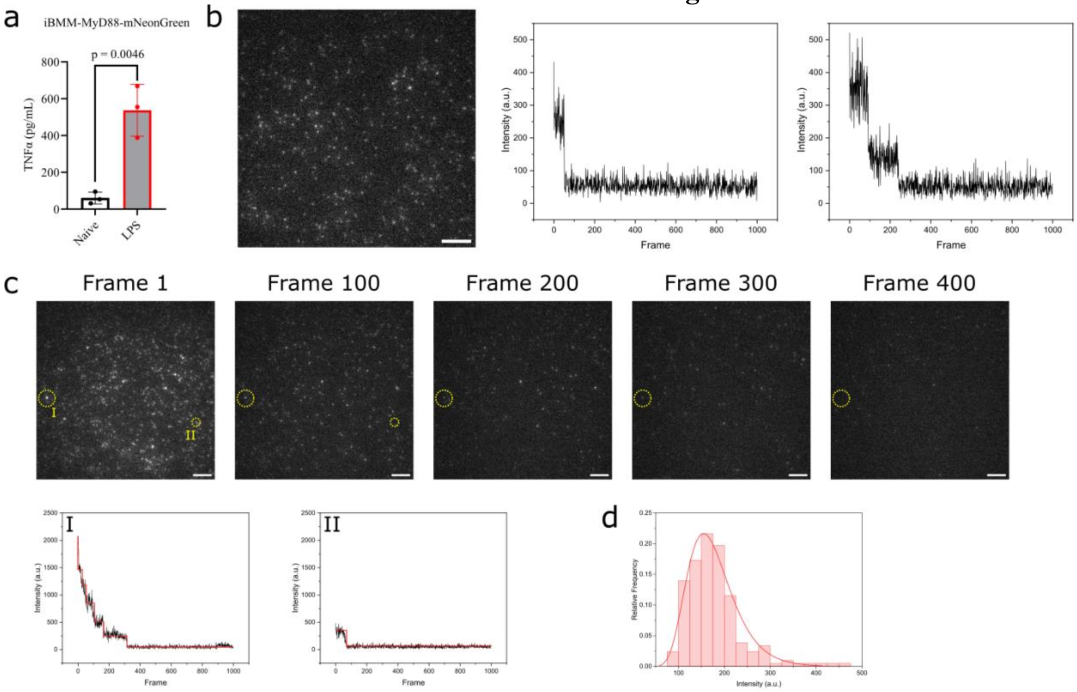

Extended Data Fig. S11

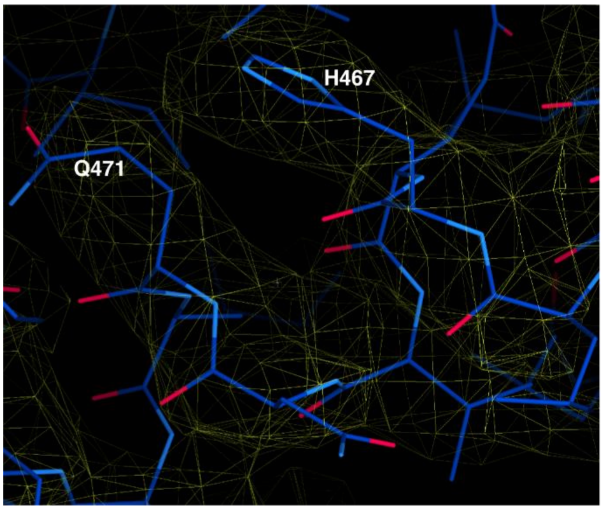

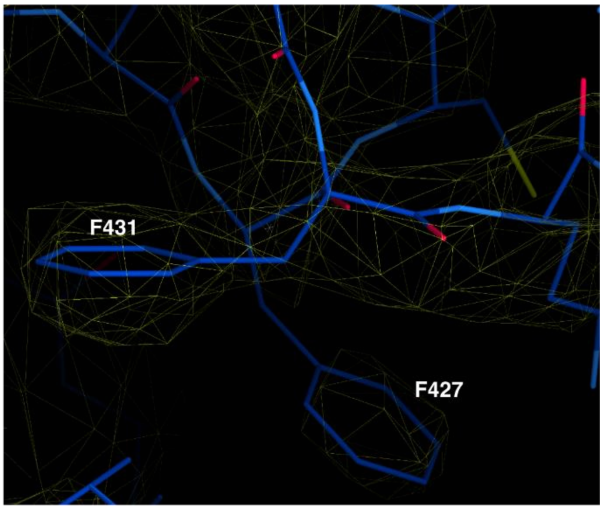

Construct	Description (human unless stated)
FL-TRIF	Full length TRIF, 1-712
FL-TRIF-Strep	Full length TRIF with streptavidin tag
RIP3-RHIM	RHIM domain of RIP3, residues 388-518.
RIP1-RHIM	RHIM domain of RIP1, residues 496-583
TRIF-R4A	Full length TRIF with alanine mutations of the core RHIM tetrapeptide ^{687}VQLG^{690}
RIP3/1-RHIM-R4A	Alanine mutations of the core RHIM tetrapeptides of RIP1 and RIP3.
TRIF-P434H	Full length TRIF with an inactivating proline to histidine mutation at 434, BB loop
TRIS-Strep	Splice variant of TRIF that deletes residues 218-658 including the TIR domain, strep tag.
TRIS-R4A	As above with the RHIM tetra peptide mutated to alanine
TRIF (153-712)	TRIF truncated at the N-terminal removing the tetratricopeptide repeat
TRIF-R4A-P434H	TRIF with alanine mutations of the RHIM core and proline to histidine mutation in the TIR domain (BB-loop)

Extended Data Table S1 – TRIF expression constructs used.

	TRIF^{TIR}
**Data Collection**	
Microscope	Titan Krios
Camera	K3
Operating voltage (kV)	300
Magnification	81,000 ± 1,620
Total exposure(e/Å^2)	53.14
Exposure per frame (e/Å^2)	1.16
Energy filter slit width (eV)	20
Pixel size (Å)	1.066
Defocus (μm)	-1 to -2.6
**Reconstruction**	
Initial number of extracted particles	8,000,000
Final number of particles	500,264
Point group symmetry	C_1
Refined helical symmetry (rise, twist)	16.1Å, 178.3°
Resolution (FSC 0.143/0.5, d_{99} Å)	3.5/4.0, 4.2
Map sharpening B-factor (Å^2)	-88
**Model composition**	
Monomer (protein residues; non-hydrogen atoms)	157; 1,239
6-mer (protein residues; non-hydrogen atoms)	942; 7,434
**Refinement and Validation (6-mer)**	
R.m.s. bond length (Å)	0.002
R.m.s angles (°)	0.6
Rotamer outliers (%)	0
Ramachandran favoured (%)	96.1
Ramachandran allowed (%)	3.9
Molprobity score	1.8
CaBLAM outliers (%)	1.3
EMRinger score	2.3
Clash score	9.9
PDB/EMDB code	8RLM/19341

Extended Data Table S2. Cryo-EM data collection, refinement and validation statistics.

Movie S1-S7

Included as separate files

Extended Data Fig. S1. Oligomeric TRIF expressed in *E. coli* binds RIP1/3. **(a)** Gel filtration profile of purified TRIF-FL (1-712) protein and SDS-PAGE analysis reveals elution of TRIF within the void volume of a S200 26/60 column. V_o = 120 mL. **(b)**, Gel filtration profile of purified TRIF-Strep. RIP1-RHIM (residues 496-583) interacts with TRIF-Strep in vitro. SE denotes the eluate from the Strep column and U denotes the unbound fraction. **(c)** TRIF-Strep binding to RHIM domains: TRIF-Strep (lane 1), RIP3-RHlM (388-518) (lanes 3-4), RIP3-RHIM-R4A mutant (lanes 5-6), RIP1-RHIM (lanes 7-8) and RIP1-RHlM-R4A mutant (lanes 9-10). U and E denote unbound and eluted fractions from the Strep column respectively.

Extended Data Fig. S2. Oligomerisation of TRIF is dependent on its TIR-domain and RHIM motif. **(a)** Negative-stain EM image of oligomeric TRIF which elutes in the void volume of a size-exclusion column reveals filaments. Scale bar: 100 nm **(b)** Limited proteolysis with subtilisin produces filaments with a different morphology which likely represent a protease resistant filament core. Scale bar: 100 nm **(c)** RHIM-independent filaments formed by the TRIF-R4A mutant are morphologically distinct and may represent oligomerisation of TRIF by the TIR domain alone. Sample filaments are indicated with blue oblongs. Scale bar: 500 nm **(d)** Possible arrangements of the amyloid fibrils in full-length TRIF. The association of exclusively odd-numbered, or exclusively even-number RHIMs produces fibrils whose long-axis is parallel to the helical axis (black cylinders). A zig-zag arrangement (grey cylinders) is produced by the association of the $i \pm n$ RHIM motifs. These fibrils are expected to lie either above or below the plane of the helical TIR-filament. The arrangement of the TIR helical filament (coloured pentagons) is identical to that depicted in **Fig 2i.**

Extended Data Fig. S3. TRIF filaments are abolished by the removal of the TIR-domain and mutations in the RHIM motif. (a) Gel filtration profiles of purified TRIF-Strep and TRIF-R4A-Strep. Both proteins elute in void volume confirming the presence of large oligomeric forms. SDS-PAGE, lane 1, Molecular weight marker; lanes 2—7 (MBP-TRIF-Strep fusion): 2, imidazole elution; 3, TEV cleaved; 4, elution after cleavage; 5, void peak; 6, unbound Strep fraction; 7, Strep elution. 8-12 are for MBP-TRlF-R4A-Strep: 8, imidazole elution; 9, imidazole elution after cleavage; 10, void peak; 11, unbound Strep fraction; 12, Strep elution. **(b)** Gel filtration profiles of purified TRIF-Strep and the BB-loop mutant TRIF-P434H showing the increase of the mutated protein in the included volume (denoted by an asterisk). The lanes of the SDS-PAGE are: 1, Molecular weight marker; 2, imidazole elution; 3, TEV cleaved; 4, elution after cleavage; 5, sample prior to gel filtration; 6—7, void peak; 8-10, gel filtration peak (*). **(c)** Gel filtration profiles of the TRIF splice variant which lacks the TIR-domain (TRIS). TRIS-Strep and TRIS-R4A-Strep show that mutation of key RHIM residues, ^{687}VQLG^{690}, disrupt RHIM fibril formation. SDS-PAGE, lanes: 2-9 (*) TRIS-Strep; 1, Molecular weight marker; 2, unbound Ni; 3, imidazole elution; 4, TEV cleaved; 5, void peak; 6, elution from Ni after cleavage; 7, unbound from Ni after cleavage; 8, unbound Strep column; 9, Strep elution. Lanes: 10-16 (**) TRIS-R4A-Strep; 10, Imidazole elution; 1 1, TEV cleaved; 12, MBP column elution; 13, concentrated protein before gel filtration; 14, gel filtration peak (209 mL); 15, unbound Strep column; 16, Strep elution.

Extended Data Fig. S4. Oligomerisation of TRIF-P434H is independent of the NTD and partially dependent on the BB-loop. (**a**) Size exclusion chromatography of TRIF-R4A-P434H and an NTD deletion mutant TRIF-(153-712)-R4AP434H on an S200 26/60 (V_0, 120 ml). SDS-PAGE, lanes: 1, Molecular weight marker; 2, Ni elution; 3, TEV cleaved; 4, gel filtration peak (119 mL); 5, gel filtration peak (153 mL); 6, gel filtration peak (190 mL). The void volume is 120 ml. (**b**) Size exclusion (S200 16/60; V_0, 47 ml) chromatography of the TRIF-R4A-BB-loop mutants.

Extended Data Fig. S5. TRIF filaments have helical symmetry. Layer lines (LL) obtained from power spectra analyses of TRIF^{TIR} filaments (**a**) and their class averages (**b**). The Bessel orders (n = 1) for LL1 and the first meridional line for which the Bessel order is zero (n = 0), which represents the helical rise are shown. (**c**) Fourier shell correlation for the reconstructed map yields a resolution of 3.5 Å (FSC = 0.143) and 3.9 Å (FSC = 0.5). The d_{99} value using the unsharpened map was 4.2 Å. Local resolution

estimates of the cryoEM map reveal that the lowest resolution regions occur at the end of helix five (αE) at the C-terminus of the TIR domain (**d**). Cryo-EM workflow used for processing the TIR filament formed by full length TRIF (**e**). The particles selected from the first classification run were subjected to a further round of 2D classification from which approximately 500,000 particles were obtained and used in helical refinement.

Extended Data Fig. S6. Model of TRIF-TIR. (a) AlphaFold model of TRIF-TIR including the extension of αE (residues 391-595, leftmost panel) and the trimmed version (residues 391-554) which was docked into the cryoEM map. The models are coloured using the predicted local distance difference test (pLDDT) scores. Regions having pLDDT scores of 100–90 are of high accuracy and include αA, αE and the ß-sheets. pLDDT scores of 90–70 include αD and parts of αC. In general, the loop regions are predicted with low confidence with the least being the ßB-loop with pLDDT scores of 70–50. Superposition of the AlphaFold (blue) and docked cryoEM (grey) models (b) and the RMSD of the C-α atoms (c).

Extended Data Fig S7. Functional validation of TRIF-mStayGold demonstrates intact TRIF-dependent signaling and cytokine responses in THP-1 cells. (a) qPCR analysis of endogenous *TRIF* mRNA expression in TRIF-mStayGold and wild-type THP-1 cells. mRNA levels were normalized to *GAPDH* and analyzed using the ΔΔCt method. Data are presented as mean ± SEM from **n = 4** independent experiments; statistical significance was assessed using an unpaired t-test. **(b, c)** Quantification of secreted TNF-α (**b**) and IFN-β (**C**) in TRIF-mStayGold and wild-type THP-1 cells following 24-hour stimulation with LPS (100 ng/mL), Pam3CSK4 (100 ng/mL), or Poly(I:C) (10 µg/mL). Data are shown as mean ± SEM from 4–5 independent experiments. **(d, e)** qPCR analysis of *TNF-α* (**d**) and *IFN-β* (**e**) mRNA expression in TRIF-mStayGold and wild-type THP-1 cells after 24-hour treatment with LPS (100 ng/mL), Pam3CSK4 (100 ng/mL), or Poly(I:C) (10 µg/mL). mRNA levels were normalized to *GAPDH* and analyzed using the ΔΔCt method. Data represent mean ± SEM from **n = 4** independent experiments. **(f)** Representative immunoblots showing NF-κB activation kinetics in wild-type and TRIF-mStayGold THP-1 cells.

Extended Data Fig. S8. Effect of IFN-β priming on gene expression and poly(I:C)-induced TNF-α secretion in TRIF-mStayGold cells. (a, b) qPCR analysis of TLR3 **(a)** and TRIF **(b)** mRNA expression in PMA differentiated TRIF-mStayGold cells either unprimed or primed with IFN-β for 24 hours. mRNA levels were normalized to *GAPDH* and analyzed using the ΔΔCt method. Data are presented as mean ± SEM from n = 4 independent experiments. **(c)** Quantification of TNF-α secretion in PMA differentiated TRIF-mStayGold THP1 cells unprimed or primed with IFN-β for 24 hours, followed by stimulation with poly(I:C) (10 µg/mL) for an additional 24 hours. Data are shown as mean ± SEM from n = 4 independent experiments.

Extended Data Fig. S9. Fluorescence analysis of TRIFosomes. (a) THP1-mStayGold cells triggered with 10 µg/mL poly(I:C) alone (without initial polarisation with IFN-β) did not induce the formation of TRIFosomes. Scale bar: 10 µm. **(b)** TRIFosome size was determined using a line profile through the major axis of identified puncta. Scale bar: 1 µm. **(c)** Gaussian fitting was performed on the intensity profiles, from which the full width at half maximum (FWHM) could be determined for the fitted function. The FWHM was used as an estimation for the diffraction-limited size of TRIF complexes. **(d)** Similar FWHM measurements were carried out on sub-diffraction 100 nm diameter beads in order to determine the FWHM of a diffraction limited object. Comparing the distribution of bead FWHMs to those of the TRIFosomes revealed that TRIF formed complexes both above and below the diffraction limit. **(e)** A small TRIFosome was identified and the containing cell subjected to photobleaching. When bleaching was nearly complete, the final 33 clear photobleaching steps were used to approximate an average intensity loss corresponding to the photobleaching of a single mStayGold fluorophore. **(f)** The initial intensity of the TRIFosome was corrected for background signal and then compared to the average step intensity. **(g)** TRIF-mStayGold cells were lysed following 100 ng/mL LPS stimulation for 90

minutes. TRIFosomes were pulled down using either a RIPK1 or IgG isotype capture antibody; no pulldown was observed in naïve cells or using the IgG isotype capture. n=3, plots indicate mean ± standard deviation, total area imaged per replicate per condition; 200,000 μm^2.

Extended Data Fig. S10 Characterization of MyD88-mNeonGreen cells and determination of MyD88 oligomeric state through photobleaching analysis. **(a)** Quantification of TNFα secretion in iBMM MyD88-mNeonGreen cells stimulated with LPS (100 ng/mL). Data are presented as mean ± SEM from three independent experiments. **(b)** Naïve MyD88 was found in a predominantly monomeric state with occasional dimers. Representative photobleaching traces are shown. Scale bar: 5 µm. **(c)** After treatment with LPS, the formation of higher order MyD88 complexes was observed. Representative photobleaching traces of a hexamer (I) and a monomer (II) are shown. Scale bar: 5 µm. **(d)** The intensity distribution of monomeric MyD88-mNeonGreen was fitted to a lognormal distribution (3 independent experiments, n = 208). The geometric mean intensity of monomeric MyD88-mNeonGreen was 169 a.u.

Extended Data Fig. S11. Side-chain fit into the cryoEM map. Typical amino acid side-chain density of subunits of the TRIF TIR filament. The density for side-chains of H467 and F431, for example, are well resolved, while Q471 is mostly resolved and F427 is poorly resolved.

Movie Legends:

Extended Movie 1:
Fluorescent signal was visible throughout the TRIF-mStayGold cell population.

Extended Movie 2:
TRIF-mStayGold dynamics in unstimulated THP1 cells visualised by TIRF microscopy. Shorter exposure times (23.5 ms) and higher illumination power densities were required to resolve the small, mostly monomeric TRIF prior to stimulation.

Extended Movie 3:
Time-lapse video illustrating the formation of TRIFosomes following LPS (100ng/ml) stimulation. Longer exposure time (100 ms) and lower illumination power densities used compared to Extended Video 2; small monomeric TRIF form a homogenous background due to the longer image exposure time, with large bright TRIFosomes resolvable over the background.

Extended Movie 4:
Time-lapse video illustrating TRIFosomes merging to form a larger complex.

Extended Movie 5:
Sequential z-sections through LPS stimulated THP1 cell acquired using epi-illumination selective plane illumination microscope (eSPIM).

Extended Movie 6:
MyD88-mNeonGreen dynamics in unstimulated iBMM cells visualised by TIRF microscopy. Shorter exposure times (20 ms) and higher illumination power densities were required to resolve the small, mostly monomeric MyD88 prior to stimulation.

Extended Movie 7:
Time-lapse video illustrating MyDDosomes merging to form a larger complex.
